# Hydroxytyrosol, Tyrosol and Derivatives and Their Potential Effects on Human Health

**DOI:** 10.3390/molecules24102001

**Published:** 2019-05-24

**Authors:** Ana Karković Marković, Jelena Torić, Monika Barbarić, Cvijeta Jakobušić Brala

**Affiliations:** Faculty of Pharmacy and Biochemistry, University of Zagreb, A. Kovačića 1, 10 000 Zagreb, Croatia; akarkovic@pharma.hr (A.K.M.); jelenatoric@gmail.com (J.T.)

**Keywords:** hydroxytyrosol, tyrosol, oleuropein, oleocanthal, oleacein, biological activities, metabolism, bioavailability

## Abstract

The Mediterranean diet and olive oil as its quintessential part are almost synonymous with a healthy way of eating and living nowadays. This kind of diet has been highly appreciated and is widely recognized for being associated with many favorable effects, such as reduced incidence of different chronic diseases and prolonged longevity. Although olive oil polyphenols present a minor fraction in the composition of olive oil, they seem to be of great importance when it comes to the health benefits, and interest in their biological and potential therapeutic effects is huge. There is a growing body of in vitro and in vivo studies, as well as intervention-based clinical trials, revealing new aspects of already known and many new, previously unknown activities and health effects of these compounds. This review summarizes recent findings regarding biological activities, metabolism and bioavailability of the major olive oil phenolic compounds—hydroxytyrosol, tyrosol, oleuropein, oleocanthal and oleacein—the most important being their antiatherogenic, cardioprotective, anticancer, neuroprotective and endocrine effects. The evidence presented in the review concludes that these phenolic compounds have great pharmacological potential, however, further studies are still required.

## 1. Introduction

The traditional Mediterranean diet (MD), characterized by regular intake of olive oil (OO), has been associated with many health benefiting effects experienced by Mediterranean populations [1]. Reduced incidence of different chronic degenerative diseases, major cardiovascular events, type 2 diabetes mellitus (DM) and some types of cancer, improved cognitive function and protection against overall morbidity and mortality are confirmed by a respectable number of trials and epidemiological studies in humans that adhere to the MD [2,3].

Consumption of virgin olive oil, a key component of the Mediterranean diet, has been recommended as a protection for high cardiovascular risk individuals [4], may prevent development of different types of cancer [5], the development of type 2 DM, obesity and metabolic syndrome [6], but also, it exerts many beneficial effects in healthy individuals [7]. There is emerging evidence that favourable health effects of MD and OO consumption could be mediated by various molecular mechanisms, one of them being the effect on the expression of genes involved in pathogenesis of many diseases and generally in aging process, according to nutrigenomic studies [7,8].

Virgin OO presents a valuable source of highly abundant unsaturated fatty acids and minor components like fat-soluble vitamins, chlorophylls, phytosterols and polyphenols [7,9]. However, when it comes to the health promoting properties, the olive oil polyphenols (OOPs) appear to be in the center of research interest [10,11,12,13]. OOPs are shown to possess antioxidant, anti-inflammatory, cardioprotective, neuroprotective, anticancer, antidiabetic, antiobesity, antisteatotic, antimicrobial and many other effects [11,14,15]. According to numerous investigations, these effects are mostly attributable to the main secoiridoid derivatives such as oleuropein (Ole), oleocanthal (Oc) and oleacein, and simple phenols hydroxytyrosol (HTyr) and tyrosol (Tyr) [7,11]. Here we review the results of the studies on biological activities, metabolism and bioavailability of the major olive oil phenolic compounds: hydroxytyrosol, tyrosol, oleuropein, oleocanthal, and oleacein, particularly regarding the new insights gained in the most recent studies.

## 2. Methodology

A systematic literature search through PubMed and Web of Science databases was carried out. The original articles investigating the biological activities of main OOPs were identified using the following search keywords: hydroxytyrosol OR tyrosol OR oleuropein OR oleocanthal OR oleacein OR olive oil phenols AND antioxidant OR anti-inflammatory OR cardioprotective OR neuroprotective OR osteoprotective OR anticancer OR antidiabetic OR antiobesity OR antimicrobial OR metabolism OR bioavailability covering the 2010–2019 time span. This large amount of literature was further manually selected according to the relevance to the topic. The focus was on the new insights regarding biological activities of named polyphenols, as well as on well established ones. Literature older than 2010 was carefully chosen and introduced when needed on the basis of its relevance to the topic.

## 3. Hydroxytyrosol

**Scheme 1:**
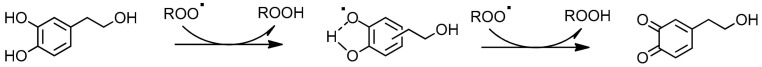
Mechanism of free radical scavenging by HTyr.

**Figure 1:**
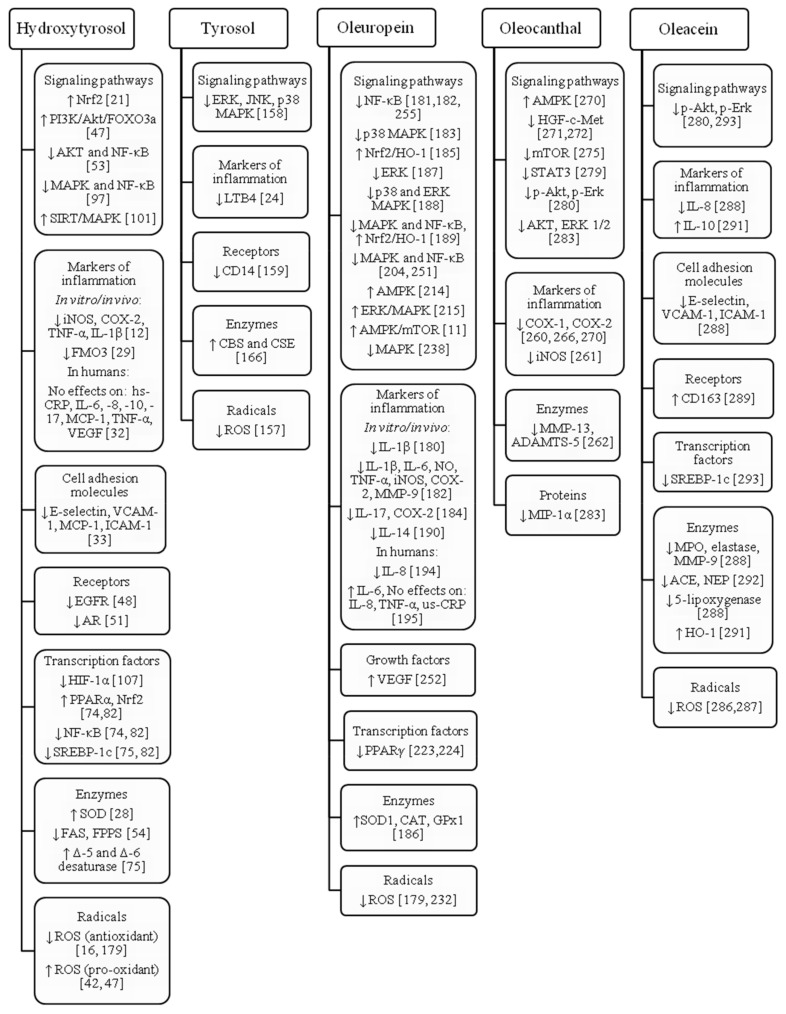
Signaling pathways and targeted molecules affected by hydroxytyrosol, tyrosol, oleuropein, oleocanthal and oleacein. ACE: angiotensin converting enzyme; ADAMTS: a disintegrin and metalloproteinase with thrombospondin motifs (aggrecanase); Akt: Protein kinase B; AMPK: adenosine monophosphate-activated protein kinase; AR: androgen receptor; CAT: catalase; CBS: cystathionine β-synthase; CD: cluster of differentiation; COX: cyclooxygenase; CSE: cystathionine *γ*-lyase; EGFR: epidermal growth factor receptor; Erk: extracellular regulated mitogen activated protein kinase; FAS: fatty acid synthase; FMO3: flavin containing monooxygenase 3; FPPS: farnesyl diphosphate synthase; GPx1: glutathione peroxidase 1; HGF/c-Met: hepatocyte growth factor/tyrosine-protein kinase Met; HIF-1α: hypoxia-inducible factor-1; HO-1: hem oxygenase 1; hs-CRP: high-sensitive C-reactive protein; ICAM-1: intracellular cell adhesion molecule 1; IL: interleukin; iNOS: inducible nitric oxide syntase; JNK: c-Jun N-terminal kinase; LTB4: leukotriene B4; MAPK: mitogen activated protein kinase; MCP-1: monocyte chemoattractant protein 1; MIP-1α: macrophage inflammatory protein 1α; MMP: matrix metalloproteinase; MPO: myeloperoxidase; mTOR: mammalian target of rapamycin; NEP: neutral endopeptidase; NF-κB: nuclear factor kappa B; NO: nitrogen oxide; Nrf2: nuclear factor E2-related factor 2; p-Akt: phosphorylated Akt; p-Erk: phosphorylated Erk; PI3K/Akt/FOXO3a: phosphoinositide 3-kinase/Akt/Forkhead box O3; PPAR: peroxisome proliferator-activated receptor; ROS: reactive oxygen species; SIRT: sirtuin; SOD: superoxide dismutase; SREBP-1c: sterol regulatory element binding protein 1c; STAT3: signal transducer and activator of transcription 3; TNF-α: tumor necrosis factor-α; VCAM-1: vascular cell adhesion molecule 1; VEGF: vascular endothelial growth factor.

Hydroxytyrosol (3,4-dihydroxyphenylethanol, HTyr), a phenolic alcohol, has been hypothesized to exert wide range of biological effects, cardioprotective, anticancer, neuroprotective antimicrobial, beneficial endocrine and other effects [10,12,16,17,18,19,20]. Although it has been extensively studied, the exact molecular mechanisms underlying many of these actions are yet to be fully clarified. Initially, the wide variety of HTyr biological activities was associated with its strong antioxidant activity (see Figure 1). HTyr acts as free radical-scavenger and metal-chelator [16]. The high antioxidant efficiency of HTyr is attributed to the presence of the *o*-dihydroxyphenyl moiety. It mainly acts as chain breaker by donating a hydrogen atom to peroxyl-radicals (ROO*). In this way fairly reactive ROO* is replaced with HTyr* radical, unreactive due to the presence of intramolecular hydrogen bond in the phenoxy radical (Scheme 1).

However, it has been proposed that HTyr may confer additional antioxidant protection by increasing the endogenous defense systems against an oxidative stress, by activating different cellular signaling pathways [21] (see Figure 1). One of the proposed mechanisms involves the HTyr-mediated induction of phase II detoxifying enzymes via nuclear factor E2-related factor 2 (Nrf2) activation in different tissues. Also, the interaction of HTyr with microRNAs (miRNAs) should be a potential molecular target for eliciting its biological effects [11,17]. miRNAs exert important regulatory actions on gene expression not only under physiological circumstances but also in disease. Indeed, miRNAs often target multiple functionally related genes and extensively interfere with biological processes, rendering them good candidates for therapeutic and/or nutraceutical interventions. HTyr modulates the expression of several miRNAs. In addition, HTyr possesses significant anti-inflammatory activity. In vitro and in vivo evidence show the attenuation of pro-inflammatory agents inducible nitric oxide synthase (iNOS), cyclooxygenase-2 (COX-2), tumor necrosis factor-α (TNF-α) and interleukin (IL)-1β expression, inhibition of granulocytes and monocytes activation by HTyr [12,22,23].

### 3.1. Antiatherogenic and Cardioprotective Effect

HTyr has been proposed to have therapeutic potential for the treatment of atherosclerosis. Atherosclerosis is considered an inflammatory disease, and the vascular endothelium is involved in many of the processes related to the development of atherosclerosis. The factors that influence endothelial function are the presence of reactive oxygen species (ROS), hypercholesterolemia, postprandial lipidemia and others [24]. Low-density lipoprotein (LDL) cholesterol oxidation is one of the key steps in the initiation of atherosclerosis. HTyr is very effective in preventing lipid peroxidation and protecting LDL from oxidation [25]. The European Food Safety Authority (EFSA) has published a health claim about the role of OOPs in protecting LDL from oxidation in vivo: “A daily intake of 20 g of olive oil, which contains at least 5 mg of hydroxytyrosol and its derivatives (e.g., oleuropein and tyrosol) provides the expected beneficial effects” [26]. HTyr exerts also beneficial effect on high-density lipoprotein (HDL) [27]. HDLs play a central role in reverse cholesterol transport, they remove excess cholesterol from peripheral cells (cholesterol efflux capacity), which predicts coronary event incidence and is inversely related to the development of early atherosclerosis. An increase in the HTyr metabolites (HTyr-sulphate, as well as homovanillic acid sulphate and glucuronate) bound to the HDLs was observed. These compounds could exert a local antioxidant protection in HDL and might prevent oxidative modifications of the apolipoprotein A-I (ApoA-I), the main HDL protein involved in cholesterol efflux capacity, and of other HDL proteins. Furtherly, it was shown that HTyr enchances the expression of cholesterol efflux related genes. HTyr is also a potential mitochondria-targeting antioxidant in the inflamed endothelium. The pretreatment of endothelial cells with HTyr suppresses inflammatory angiogenesis, reduces mitochondrial superoxide production and lipid peroxidation and increases superoxide dismutase (SOD) activity [28]. It also exerts beneficial effects in atherosclerososis by its anti-inflammatory activity. HTyr can alleviate the inflammatory response of flavin containing monooxygenase 3, the enzyme that may drive the development of atherosclerosis, in cells treated with highly toxic air pollutant acrolein [29]. In vivo evidence confirmed that HTyr administration prevented LPS (lypopolisaccharide)-induced effects and improved the antioxidant power of plasma [30]. Clinical trial has shown that regular intake of 15 mg/day of HTyr changed body composition parameters and modulated the antioxidant profile, the expression of inflammation and oxidative stress-related genes in atherosclerosis [31]. On the other hand, in another trial with healthy volunteers, consumption of olive mill wastewater extract selectively enriched in two HTyr doses (5 and 25 mg/day) for 1 week resulted in no significant effects on inflammation markers, such as high-sensitive C-reactive protein (hs-CRP), interleukin 6 (IL-6), IL-8, IL-10, IL-17, monocyte chemoattractant protein 1 (MCP-1), tumor necrosis factor alpha (TNF-α) and vascular endothelial growth factor (VEGF) [32]. Furtherly, HTyr exerted a vascular protective effect through the down-regulation of vascular cell adhesion molecules involved in early atherogenesis [22]. Feeding ApoE−/− mice with 10 mg/kg/day of HTyr derivatives for 12 weeks significantly reduced E-selectin, vascular cell adhesion molecule-1 (VCAM-1), MCP-1, intracellular cell adhesion molecule-1 (ICAM-1) expression and F4/80 macrophage marker expression compared with the control group, in the isolated aorta [33]. Postprandial lipemia presents a risk factor for atherosclerosis development and it was reported that HTyr improves blood lipids profile, due to its ability to lower serum total cholesterol (TC), triglycerides (TG), and LDL levels [34]. Supplementation of olive mill waste water extract (5 and 25 mg HTyr/day for 1 week) to healthy volunteers [32] resulted in the absence of significant effects on blood lipid parameters [35]. In the other trail HTyr was administered at a daily dosage of 45 mg for 8 weeks to volunteers with mild hyperlipidemia. This dose did not influence blood lipids, but endogenous vitamin C levels were increased [36]. Moreover, HTyr-nicotinate, HTyr-clofibrate and HTyr-fenofibrate showed the antihyperlipidemic effect [37,38,39]. HTyr and HTyr-acetate (HTyr-Ac) were proved to prevent platelet aggregation, one of the factors involved in thrombotic processes, with effects similar to acetylsalicylic acid [40]. It was also shown that HTyr has cardioprotective activity by down-regulating proteins related to proliferation and migration of endothelial cells and occlusion of blood vessels in aorta and proteins related to heart failure in heart tissue [41].

### 3.2. Anticancer Effects

Over the last decade, a vast number of in vitro and in vivo studies have shown significant anticancer effects of HTyr against various types of malignant cells, with different mechanisms of action being proposed [11,12,42,43]. Much of the research was focused on colon cancer, the third most common cancer worldwide, with rising incidence and mortality in developing countries. HTyr, although present in relatively low concentrations in OO, reaches significant levels in large intestine due to the gastric hydrolysis and colonic fermentation of secoiridoids present in OO [44]. Together with its active metabolites, HTyr is likely to be a major candidate for the noticed biological activity of OOPs on human colon adenocarcinoma cells [45,46]. Accumulation of H_2_O_2_ probably due to its autooxidation certainly presents the most common anticancer mechanism of HTyr [42]. However, numerous studies highlighted different antiproliferative and pro-apoptotic mechanisms of HTyr depending on the cancer cell type studied [11]. Up till now, the beneficial anticancer effects of HTyr were investigated in colorectal, breast, bladder, blood, gastric, hepatic, skin, prostate, cervical, brain, lung and thyroid types of cancer suggesting its possible wide use in cancer prevention and treatment [43].

HTyr had an antiproliferative effect and induced apoptotic cell death through ROS generation which was followed by activation of PI3K/Akt/FOXO3a pathway and a decrease in the antioxidant defense capacity in DLD1 human colon cancer cells [47]. Antiproliferative and proapoptotic effect of the HTyr via activation of caspase signaling was shown in colon cancer cells Caco-2 and HT-29 [46]. Furthermore, it was demonstrated that HTyr significantly down-regulates epidermal growth factor receptor (EGFR) expression, which was associated with reduced cell proliferation of human colon cancer cells and decreased tumor growth and EGFR expression levels in HT-29 xenografts [48]. Lipophilized natural extracts containing HTyr showed an antiproliferative effect on the human colon cancer cells and the greatest effect was that of HTyr-oleate fraction, probably due to the major lipophilicity of the oleate chain [49].

The investigation of the effect of HTyr on breast, prostate and colon cancer cell lines revealed that the antiproliferative activity of HTyr was inversely correlated to the ability of the different cell lines to remove H_2_O_2_ from the culture medium, with the prostate cancer cells being more resistant to the growth inhibition than the breast and colon cancer cells [50]. Furthermore, the investigation on androgen-dependent (AR) prostate cancer cells showed that HTyr inhibits AR expression and the secretion of AR-responsive prostate-specific antigen [51]. The effect of HTyr on the oxidative stress response of the human breast cancer cell line MCF-7 in hypoxia and normoxia was investigated. It was shown that HTyr is particularly effective in a hypoxic environment. Although the transcriptional effects of HTyr were similar in normoxic and hypoxic conditions, its translational action was less prominent and partially attenuated in hypoxia [52].

The anticancer effects of HTyr in human hepatocellular carcinoma (HCC) cells were explored with results showing inhibition of proliferation, induction of G2/M cell cycle arrest and apoptosis in vitro. Inhibition of the tumor growth and angiogenesis was demonstrated in vivo, by suppression of the activation of protein kinase B (Akt) and nuclear factor-kappa B (NF-κB) pathways [53]. HTyr showed the antiproliferative and proapoptotic effects by inhibiting the lipogenic enzymes fatty acid synthase (FAS) and farnesyl diphosphate synthase (FPPS) in human hepatoma cells, whose higher levels may be related with aggressive behavior of tumor [54]. Furthermore, HTyr inhibited the proliferation of the human cholangiocarcinoma (CCA) and human gallbladder cancer cell lines, and markedly inhibited the growth of CCA xenografts in mice [55]. HTyr arrested the cell cycle, increased the Bax/Bcl-2 ratio, increased activation of caspase 3/7 and induced apoptosis in pancreatic MIA PaCa-2 cancer cells [56].

The first study of anticancer properties of HTyr in human differentiated thyroid carcinoma showed that HTyr increased apoptosis via activation of mitochondrial apoptotic mechanism in papillary and follicular cancer cells. Concurrently, the strong resistance of thyroid cancer cells to the HTyr was shown, with higher doses needed for the similar antiproliferative effect in comparison to some other types of cancer cells, e.g., colon or breast ones [57].

### 3.3. Neuroprotective Effects

The neuroprotective effects of HTyr were studied in a great number of in vitro and ex vivo studies by different strategies, some of them employed chemically induced neurotoxicity or were based on the biochemical alterations that take place during the process of hypoxia-reoxygenation [10]. The brain accumulation of HTyr and HTyr-sulphate suggested their neuroprotective activity by the reduction of the oxidative stress at neuronal level [58]. One of the main age-associated neurodegenerative diseases is Alzheimer’s disease (AD), an amyloid disease characterized by the deposition of typically aggregated protein/peptides in tissues that are associated with brain degeneration and progressive cognitive impairment. HTyr has been studied in vitro in AD models. It protected neuronal cells against amyloid-β induced toxicity and prevented tau fibrillization [10]. HTyr was an effective inhibitor of hen egg white lysozyme aggregation, thus suggesting possible future applications of this natural compound for prevention or treatment of amyloid diseases [59].

HTyr is a promising compound for Parkinson’s disease (PD) medication, as it inhibits both enzymatic and spontaneous oxidation of endogenous dopamine, mitigates the increase in spontaneous oxidation during MAO inhibition, has a protective effect against dopamine and 6-hydroxydopamine (6-OHDA)-induced dopaminergic cell death and counteract α-synuclein-induced toxicity [60,61]. HTyr-butyrate also inhibits 6-OHDA-induced apoptosis through activation of the Nrf2/HO-1 (heme oxygenase 1) axis in neuronal cells [62].

In vivo studies have shown that HTyr attenuates the spatio-cognitive deficits, restores learning capacity and memory performance, and promotes cognitive function as well [63,64]. Also, the combination of HTyr and oleic acid was shown to inhibit cholesterol and fatty acid synthesis in C6 glioma cells [65]. HTyr may confer protection against ethanol-induced oxidative stress [66].

### 3.4. Antidiabetic, Lipid-Regulating and Antiobesity Effects

HTyr demonstrates hypoglycemic activity in various diabetic animal models by influencing the major biochemical processes leading to diabetic vasculopathy [67,68]. An animal model experiments showed that HTyr could effectively prevent diabetic neuropathy [69,70]. It was shown that HTyr improves insulin sensitivity and restores proper insulin-signaling [71].

HTyr exerts a protective effect on the liver [72], particularly on non-alcoholic fatty liver disease (NAFLD), a condition characterized by an increment in the liver fat content, with a concomitant reduction in the content of *n*-3-long chain polyunsaturated fatty acids (LCPUFA), and the inflammatory progression to steatohepatitis. Feeding animals with a high fat diet (HFD) is considered adequate experimental model to study interventions for preventing or treating NAFLD. HTyr significantly improves metabolic impairments induced by HFD [73,74]. HTyr prevents early inflammatory events responsible for the onset of insulin resistance and steatosis, by reducing the hepatic inflammation and nitrosative/oxidative stress and restoring glucose homeostasis and intestinal barrier integrity [73]. It activates transcription factors as peroxisome proliferator-activated receptor (PPAR)-α and Nrf2, and deactivates NF-κB [74]. Another study has shown that protective effects of HTyr on the liver are associated with the recovery of the activity of Δ-5 and Δ-6 desaturase enzymes, the most relevant enzymes in biosynthesis of LCPUFAs (eicosapentaenoic acid, EPA, C20:5n-3, docosahexaenoic acid, DHA, C22:6n-3 and arachidonic acid, AA, C20:4n-6), downregulation of the lipogenic factor sterol regulatory element binding protein (SREBP)-1c and the maintenance of the levels of n-3 LCPUFAs in extrahepatic tissues [75]. The effects of combined EPA (50 mg/kg/day) and HTyr (5 mg/kg/day) were studied as a potential therapy for NAFLD and it was shown that this combination drastically decreases NAFLD development, mostly due to the effect of HTyr [76].

HTyr inhibits lipogenesis, both *de novo* fatty acids and cholesterol syntheses without an effect on cell viability and the metabolism of linoleic acid, retinol, sphingolipids and arachidonic acid, whereas glycerolipid metabolism is upregulated [77]. It was shown that HTyr has beneficial effect in hyperlipidemia as it modifies genes related with adipocyte maturation and differentiation and inhibits lipid formation [78]. HTyr was shown to be effective towards the mobilization of lipids [79]. Also, HTyr has beneficial effect on obesity by suppressing dose-dependently intracellular TG accumulation and the expression of adipogenesis-stimulating factors during adipocyte differentiation, promotes lipolysis in human primary visceral adipocytes during differentiation and decreases hepatic steatosis [80,81]. HTyr supplementation improves the white adipose tissue dysfunction induced by HFD in mice through the modulation of transcription factors NF-κB, Nrf2, SREBP-1c, and PPAR-γ as well as their target genes [82].

HTyr acts as caloric restriction (CR) mimicker. The effectiveness of CR to prolong lifespan and to reduce the risk of age-associated diseases is widely recognized. However, a CR regime can hardly be sustained for long periods of time; this is why diet integration with factors able to mimic the beneficial effects of a reduction of caloric intake can be highly appreciated. HTyr induces CR-like effects in muscle, brain, fat tissue and kidney in several ways, particularly through the activation and increased levels of sirtuins, whose activity is modulated by the metabolic state of the cells and induced by CR [11].

### 3.5. Antimicrobial and Antiviral Effects

It was demonstrated that HTyr possesses in vitro antimicrobial properties against infectious agents of the respiratory and gastrointestinal tracts such as *Vibrio parahaemolyticus*, *Vibrio cholerae*, *Salmonella typhi*, *Haemophilus influenzae*, *Staphylococcus aureus*, or *Moraxella catarrhalis*, at low inhibitory concentrations [83], and also against beneficial microorganisms such as *Lactobacillus acidophilus* and *Bifidobacterium bifidum* and foodborne pathogens as *Listeria monocytogenes*, *Staphylococcus aureus*, *Salmonella enterica*, *Yersinia* [84]. Antimicrobial activity against *Staphylococcus aureus* and *Staphylococcus epidermidis* was evaluated in HTyr-Ac and HTyr-oleate compounds as well [85,86]. HTyr inhibits hemolytic activity induced by streptolysin O that is produced by *Streptococcus pyogenes* [87]. Moreover, HTyr has an antibacterial effect against *Propionibacterium acnes* [88]. HTyr is also effective against mycoplasmas such as *Mycoplasma pneumoniae* [89]. Contrary, one study indicates that HTyr did not show any significant antimicrobial activity [90].

HTyr was identified as a unique class of HIV-1 inhibitors that prevent HIV from entering into the host cell and binding the catalytic site of the HIV-1, with both, viral entry and integration inhibited [91]. Furthermore, it was reported that HTyr inactivated influenza A viruses, suggesting that the mechanism of the antiviral effect of HTyr might require the presence of a viral envelope [92].

The antifungal activity of a series of chemically synthesized HTyr analogs was investigated. Using *Aspergillus nidulans* as an in vivo cellular model system, it was shown that antifungal HTyr analogs have an unprecedented efficiency in fungal plasma membrane destruction [93]. It was demonstrated that HTyr has antitrypanosomal and antileishmanial activity [94]. HTyr alkylcarbonate derivatives were active against *Trypanosoma brucei* [95]. Trypanosomiasis and leishmaniasis, infectious diseases caused by protozoan parasites, affect millions of people around the world, especially in tropical and subtropical area, and present one of the major causes of hunger and poverty in sub-Sahara Africa.

### 3.6. Other Effects

A beneficial role of HTyr in several inflammatory diseases was proposed [12,96]. HTyr may be advantageous in rheumatoid arthritis, autoimmune disease characterized by chronic inflammation, with significant impact not only on chronic inflammation but also on acute inflammatory processes. Protective effects could be related to the inhibition of mitogen-activated protein kinase (MAPK) and NF-κB signalling pathways [97]. HTyr has the potential as a chondroprotective compound against osteoarthritis (OA), as an autophagy and sirtuin-1 (SIRT1) inducer [98]. HTyr and HTyr-Ac reduced pro-inflammatory cytokines and prevented renal damage with a considerably blockage of different inflammatory-related pathways suggesting that HTyr and HTyr-Ac supplementation might provide a basis for developing a new dietary strategy for prevention and management of systemic lupus erythematosus [99]. It was shown that HTyr-Ac exerts an anti-inflammatory effect on acute ulcerative colitis [100]. In vivo evidence supported the protective effect of HTyr on lung inflammation, attributed to the promotion of autophagy, which is associated with the activation of the SIRT/MAPK signaling pathway [101].

Furthermore, HTyr shows osteoprotective effects. It has beneficial effects on the formation and maintenance of bone, as it can stimulate the deposition of calcium, inhibit the formation of multinucleated osteoclasts in a dose-dependent manner, and suppress the bone loss of spongy bone, as was observed in femurs of ovariectomized mice [102]. Also, HTyr stimulates human osteoblastic cell proliferation [103].

HTyr modulated allergen Par j 1-induced IL-10 production by peripheral blood mononuclear cells in healthy subjects, demonstrating its ability to affect an allergen-specific immune response by potentiating a suppressive immune response towards an allergen [104].

It was shown that HTyr has protective effect on UV-B irradiated keratinocytes [105,106]. UVB radiation represents the major cause of serious skin disorders and genotoxic damage. The long-term exposure to light-emitting-diode-generated blue light (LED-BL) from electronic devices seems to have a relevant implication in the molecular mechanisms of premature photoaging.

Beneficial effect of HTyr in renal hypoxia was suggested [107]. HTyr treatment decreased both, posthypoxic ROS and NO levels and consequently, hypoxia-inducible factor-1 (HIF-1) accumulation, and promoted the expression of angiogenic proteins, suggesting that HTyr activates an adaptive response to hypoxia in a HIF-1α-independent pathway that could be ascribed to the up-regulation of estrogen-related receptor α.

### 3.7. Metabolism

The bioavailability and excretion of HTyr were first determined in humans by Visioli et al. [108]. The poor bioavailability of HTyr observed in different studies related to the low or null concentration of circulating HTyr is due to the subsequent extensive first-pass phase-I and phase-II metabolism in the gut and liver [109]. Some studies described that gender is a critical feature for the final bioavailability of HTyr. It was shown that after oral administration of HTyr and its precursors from OO, this compound persists for a longer time in the body of female rats [19,110]. Therefore, HTyr precursors (Ole, oleacein and Ole aglycone) are mainly hydrolysed during gastrointestinal digestion, giving finally HTyr as their metabolite [110].

The polar character of HTyr hinders its solubility in lipid media and impedes it from passing across the bilayer membrane at the intestinal level [58]. In recent years, various lipophilic compounds derived from HTyr have been synthesized to improve its pharmacological activity and new lipophilic antioxidants for food applications were developed [111]. HTyr-Ac, the most widely studied lipophilic derivative of HTyr, which can also be found naturally in virgin OO at low concentrations, can cross the intestinal barrier easily [112]. Other synthetic HTyr ether derivatives with antioxidant and anti-inflammatory activity have shown an efficient absorption, showing different intestinal transport rates in accordance with their lipophilicity (butyl > propyl > ethyl > methyl) [113]. The alkyl chain length influences their bioactivity, in a manner that short to medium length chains possess even higher antioxidant activity than HTyr, whereas long chains reduce their bioactivity [58,114,115]. HTyr is normally absorbed in the intestine through bi-directional passive diffusion mechanism [43,116] with an efficiency that oscillates from 75% up to 100% [19]. The absorption process depends on the composition of the food matrix through which HTyr is administered. Stability of HTyr under digestion conditions is higher after its administration as a natural component of OO, especially extra virgin OO [117], than after its addition to a water vehicle or refined OO [19,109,118,119,120]. Some studies found that HTyr has a plasma half-life of 1–2 min [121]. Once absorbed, HTyr quickly incorporates in plasmatic HDLs, acting as an antioxidant and as a cardiovascular protector [19,122].

HTyr and its metabolites have very good distribution abilities in tissues such as muscle, testis, liver, and brain; besides they are accumulated in kidney and liver [121,123]. Due to the fact that HTyr is found in brain tissue (due to its ability to cross the blood-brain barrier) and because of its possible interaction with dopaminergic pathways, HTyr is considered to be a dopaminergic neuronal protector [124,125]. This widespread distribution is responsible for the health beneficial properties of HTyr [123].

**Scheme 2:**
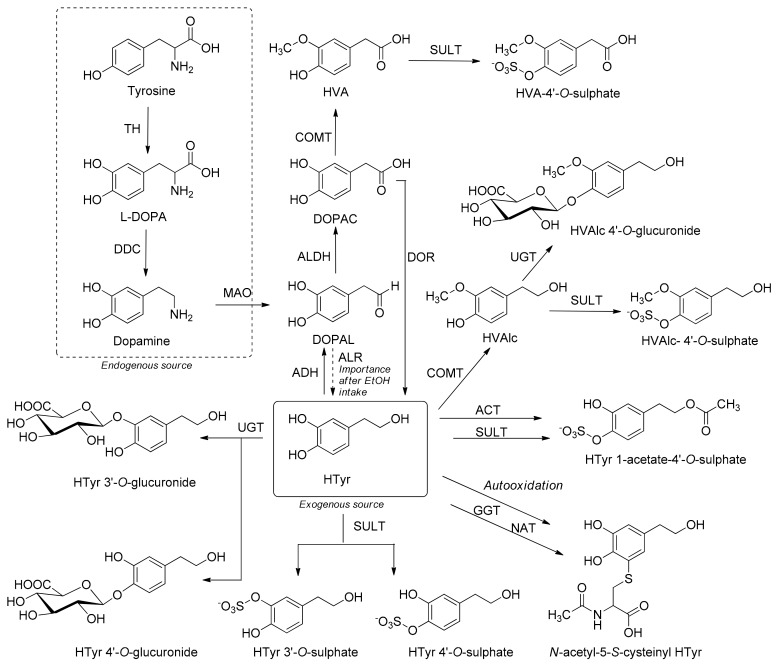
Metabolic pathways of endogenous and exogenous HTyr. HVAlc: homovanillic alcohol; HVA: homovanillic acid; EtOH: ethanol; TH: tyrosine hydroxylase; DDC: dopa decarboxylase; MAO: monoaminoxidase; ALDH: aldehyde dehydrogenase; ALR: aldehyde/aldosa reductase; ADH: alcohol dehydrogenase; DOR: DOPAC reductase; COMT: catechol-O-methyltransferase; UGT: uridine 5′-diphosphoglucuronosyl transferases; SULT: sulphotransferase; ACT: *O*-acetyltransferase; GGT: γ-glutamyl transpeptidase; NAT: *N*-acetyl transferase.

The first step of its intense and rapid metabolism happens inside enterocytes and subsequently in the liver [19]. The enzymes involved in HTyr phase-I metabolism, mostly present in the intestinal wall, are non-microsomal alcohol and aldehyde dehydrogenases (ALDH), both located in the cytosol [126]. HTyr is naturally present in the brain as by-product of dopamine and tyramine metabolism [126,127]. Deamination of dopamine by monoaminoxidase (MAO) produces 3,4-dihydroxy-phenylacetaldehyde (DOPAL), an unstable and toxic oxidation product that can be easily oxidized by ALDH to the acidic metabolite 3,4-dihydroxyphenylacetic acid (DOPAC) [109,121]. In a lesser amount, DOPAL is reduced to HTyr by the aldehyde/aldose reductase (ALR) and HTyr can reversely return to DOPAL mediated by alcohol dehydrogenase (ADH). Furthermore, DOPAC can be transformed into HTyr by DOPAC reductase [128]. Some studies also suggest that the metabolization of ethanol might block enzymes ADH and ALDH, which could increase the levels of HTyr in the circulation [109,129] by the transformation of DOPAC into HTyr [128,130,131,132,133] or reduction DOPAL by ALR into HTyr [129] (Scheme 2).

The enzymes involved in HTyr phase-II reactions, sulphotransferases (SULT), uridine 5′-diphosphoglucuronosyl transferases (UGT) and catechol-*O*-methyltransferase (COMT), form the main HTyr metabolites detected in biological samples [119,123,134,135,136,137]. The metabolites include *O*-methylated forms: homovanillic acid (HVA) and homovanillyl alcohol (HVAlc) which are brought about by the action of COMT to HTyr and the metabolite DOPAC, respectively [138]. It seems that under alkaline conditions in the human lumen, HTyr can also be a substrate of enzyme acyltransferase which catalyses the transfer of acetyl group from acetyl-CoA to HTyr and forms metabolite HTyr 1-acetate. This metabolite can be biotransformed into HTyr 1-acetate-4′-*O*-sulphate by enzyme SULT [109,134]. The metabolite *N*-acetyl-5-*S*-cysteinyl HTyr starts from the autoxidation of HTyr to HTyr quinone. In the further reaction with glutathione (GSH), it produces the conjugate, which is cleaved to the final metabolite with the enzymes γ-glutamyl transpeptidase (GGT) and *N*-acetyl transferase (NAT). The mercapturate conjugate of HTyr is formed in a dose-dependent manner [139]. HTyr sulphate is the main circulating metabolite detected in rat plasma [123,136] whereas in humans, HTyr-sulphate and HTyr 1-acetate-4′-*O*-sulphate present the main metabolites detected in plasma after the consumption of HTyr or HTyr derivatives at normal dietary doses [109,121,134,137,140,141] (Scheme 2). In most of the in vitro studies the presence of methyl/sulfate/glucuronide functional groups did not seem to inhibit their biological activity [142].

**Scheme 3:**
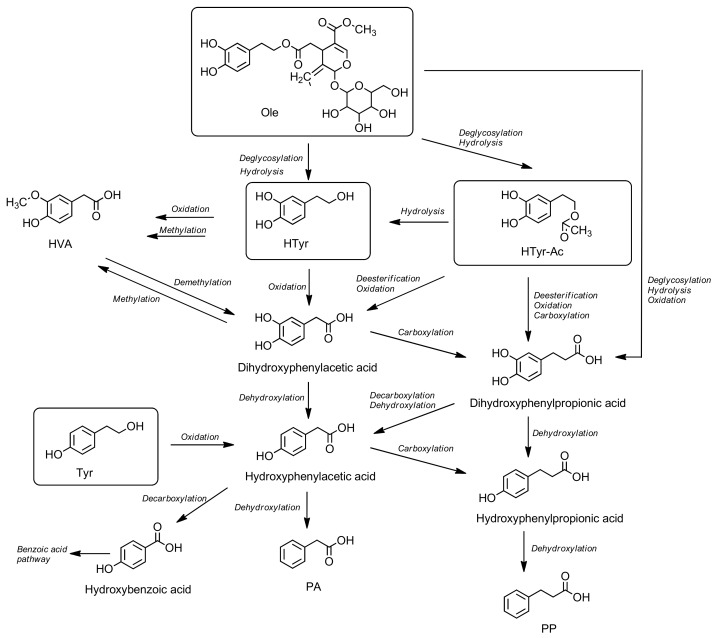
Proposed colonic pathway of hydroxytyrosol (HTyr), tyrosol (Tyr), hydroxytyrosol acetate (HTyr-Ac) and oleuropein (Ole). HVA: homovanillic acid; PA: phenylacetic acid; PP: phenylpropionic acid.

Over recent years, the behaviour of phenolic compounds in the colon has attracted much attention as most phenolic compounds pass through the small intestine without being absorbed, thus encountering the gut microbiota which may have an impact in humans [109]. This has led to the development of two-way mutual reactions between phenolic compounds and gut microbiota. First, the non-digested phenolic compounds are biotransformed into their catabolic metabolites by gut microbiota, and this results in an increase of their bioavailability [109,143]. The microbiota transforms unmetabolized native phenols from OO into different catabolites mainly by oxidation and dehydroxylation reactions. In the intestine and cecum, HTyr is thoroughly transformed by microbiota into phenylacetic acid (PA) and its derivatives; however, the catabolism of HTyr-Ac produces phenylpropionic (PP) derivatives. Then, these colonic metabolites could be absorbed and subsequently transformed into a phase-II metabolites [13,46,144,145] (Scheme 3). Secondly, the phenolic compounds themselves modulate the composition of the gut microbial community, mostly through the inhibition of pathogenic bacteria and the stimulation of beneficial bacteria such as bifidobacteria acting as prebiotics [109,146,147].

Taking into account the structure and hydrophilic properties of HTyr molecule and its intense metabolism and transformations, its conjugated metabolites are mainly excreted by the kidneys [119]. The time required for the complete elimination from the body, both for HTyr and its metabolites, is approximately 6 h in humans [126,148] and around 4 h in rats [110]. HTyr is accumulated in the kidneys until its excretion [121] where it may perform a nephroprotective role due to its antioxidant properties [149]. The biliary route redirects HTyr metabolites from the liver back into the duodenum where they can be transformed and reabsorbed. Thus, this enterohepatic recycling may lead to a longer presence of HTyr and metabolites within the body [109]. Around 5% of total HTyr is excreted by faeces 5 h after an injection of HTyr [19,121]. The future perspectives on the HTyr research will be to study the effects of the metabolites because the concentrations of HTyr in biological fluids are extremely low compared to their metabolites [117,120,139,150,151]. At present, very few studies have tested the bioactivity of HTyr metabolites [142,152,153,154], but it seems probable to assume they are bioactive and responsible to a great extent for HTyr biological effects [109].

To corroborate the previous assumption, the antioxidant activities of HTyr glucuronides were reconsidered within a range of concentrations compatible with their dietary consumption [126,152]. The glucuronide forms of HTyr neutralized the endoplasmic reticulum oxidative stress in the HepG2 cell line related to the development of cardiovascular diseases [153]. A mixture of HTyr metabolites biologically obtained by the exposure of HTyr to Caco-2 cells (80% HTyr-sulphate) showed a significant effect on the decrease of inflammation biomarkers in human aortic endothelial cells (HAEC) leading to an improvement in the endothelial dysfunction [41]. HTyr-3′-*O*-glucuronide and hydroxytyrosol-4′-*O*-glucuronide also protected against the induced hemolysis by H_2_O_2_ in red blood cells in a dose-dependent manner [155]. By contrast, in a porcine kidney epithelial cell line, co-incubation with HTyr-3′-*O*-glucuronide and HTyr-4′-*O*-glucuronide did not inhibit oxidative damage and cell death induced by H_2_O_2_. However, pre-incubation with 10 µM of HTyr-glucuronides protected against tubular membrane oxidative damage like native HTyr did and also diminished the formation of malondialdehyde, fatty acid hydroperoxides and 7-ketocholesterol [109,156]. A recent report found that HTyr and Tyr sulphates displayed efficiency in protecting the oxidative damage by ox-LDL in Caco-2 cells compared to that of the parent compounds [109,126,154].

## 4. Tyrosol

Tyrosol (2-(4-hydroxyphenyl)-ethanol, Tyr), was shown to be effective cellular antioxidant, probably due to intracellular accumulation, in spite of its weak antioxidant activity [157]. Tyr is a rather stable compound and therefore, when compared with other polyphenols, much less subject to autooxidation. It maintains antioxidant activity under critical conditions as well. In the presence of oxidized LDL, when autoxidation phenomena had already started, Tyr maintained an unchanged antioxidant activity, while other, more active natural flavonoids showed a drastic reduction of their antioxidant activity and sometimes even became pro-oxidants [24]. It was shown that Tyr is effective in inhibiting oxidative damage of L6 muscle cells by inhibiting H_2_O_2_-induced cell death in part through regulation of extracellular signal-regulated kinases (ERK), c-Jun *N*-terminal kinase (JNK), and p38 MAPK and by increasing ATP production [158] (see Figure 1). The effects of Tyr on modulation of the upstream pathways regulating LPS-induced inflammatory response were investigated. Study confirmed that Tyr could exert its beneficial effects against hypertension, atherosclerosis, coronary heart disease, chronic heart failure, insulin resistance and obesity by modulating cluster of differentiation 14 (CD14) up-regulation and inhibiting inflammation [159]. Tyr reduces oxidative modifications to HDL and by maintaining the physicochemical properties of HDL, improves the functionality of HDL, especially the capacity to promote cholesterol efflux [27]. It also posseses antiatherogenic activity due to inhibition of leukotriene B4 production that affects endothelial function [24]. Furtherly, it was shown that Tyr induces myocardial protection against ischemia related stress and could be considered as anti-aging therapy [160]. Today it is clear there is a need for new therapeutic strategies in the treatment of arterial hypertension that should take into account not only the level of blood pressure (BP), but also other risk factors that lead to the development of cardiovascular complications, as hyperviscosity syndrome. One such strategy may be the correction of microcirculatory disturbances by affecting the rheological properties of the blood. Tyr has been evaluated as promising substance that affects microcirculation, taking into account its hemorheological activity. For example, experiments in vitro showed that Tyr constrains the increase in blood viscosity and the effect of Tyr was comparable to that of pentoxyphylline. Also, the effect of Tyr on hemorheological parameters and cerebral microvascularization in spontaneously hypertensive rats was investigated at the stage of BP rising, and it was shown that Tyr limits the development of hyperviscosity syndrome, improves oxygen transport capacity and eliminates microvascular rarefaction in the cerebral cortex [161]. In acute cerebral ischemia-reperfusion Tyr reduces the blood viscosity and the intensity of oxidative stress in the brain tissue [162]. Tyr treatment ameliorated hyperglycemia by regulating key enzymes of carbohydrate metabolism in streptozotocin-induced diabetic rats. Tyr may also play an important role in the treatment of DM, as it exerts anti-inflammatory effects on the liver and pancreas of streptozotocin-induced diabetic rats [163], and inhibits endoplasmatic reticulum stress-induced apoptosis in pancreatic β-cell [164]; dysfunction of pancreatic β-cells is a major determinant for the development of type 2-DM. It was shown that Tyr down-regulated lipid synthesis in primary-cultured rat-hepatocytes [165]. Tyr also exerts beneficial effect in NAFLD, where oxidative stress is one of the important factors responsible for the development and progression of disease [166]. Hydrogen sulfide (H_2_S), a multifaceted gasotransmitter, has emerged as a potential therapeutic target in NAFLD and cystathionine β-synthase (CBS) and cystathionine γ-lyase (CSE) are major enzymes responsible for endogenous H_2_S synthesis. Tyr supplementation significantly increased hepatic CBS and CSE expression and H_2_S synthesis in HFD-fed mice. Tyr has neuroprotective effect against Aß-induced toxicity in AD [167]. Also, it possesses potent activity against several strains of bacteria responsible for intestinal and respiratory infections in vitro [84]. Antibacterial properties of Tyr can be linked to the binding and inhibition of bacterial ATP synthase [168]. Antitrypanosomal and antileishmanial activities of Tyr were also observed [94].

Tyr prevented osteopenia by increasing bone formation, probably because of its antioxidant properties [169]. Furthermore, Tyr showed antigenotoxic activity and could prevent apoptosis in keratinocyte [105]. The effect of Tyr on metazoan *Caenorhabditis elegans* longevity was investigated. Tyr treatment extended mean and maximum lifespan of *C. elegans*, increased thermal and oxidative stress resistance and slowed the aging rate. It was proposed that factors of the heat shock response might mediate the effects of Tyr on longevity [170].

### Metabolism

Tyr is exposed to an extensive metabolism in the human body, and its bioavailability is poor with respect to its metabolites [126,142]. The results of the study in rats indicate that Tyr is rapidly absorbed and excreted via the kidney within 8 h after oral administration [171]. Secoiridoids which contain Tyr (ligstroside, Oc) are subject to time-dependent hydrolysis in the acidic gastric environment, leading to an approximate 3-fold increase in free Tyr after only 30 min [44].

**Scheme 4:**
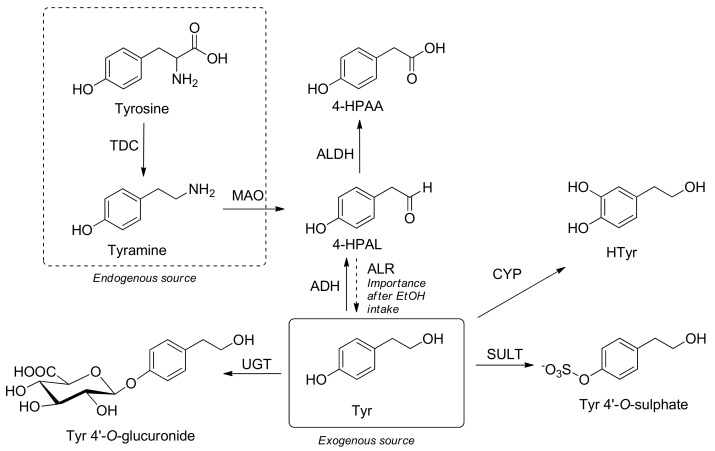
Metabolic pathways of endogenous and exogenous tyrosol (Tyr): Htyr: hydroxytyrosol; 4-HPAA: 4-hydroxyphenylacetic acid; 4-HPAL: 4-hydroxyphenylacetaldehyde; EtOH: ethanol; TDC: tyrosine decarboxylase; MAO: monoaminoxidase; ALDH: aldehyde dehydrogenase; ALR: aldehyde/aldosa reductase; ADH: alcohol dehydrogenase; CYP: cytochrome P450; UGT: uridine 5′-diphosphoglucuronosyl transferases; SULT: sulphotransferase.

The endogenous Tyr is formed by oxidative metabolism of tyramine which is a monoamine compound derived from the decarboxylation of the amino acid tyrosine through tyrosine decarboxylase (TDC). In a similar way to other biogenic amines, tyramine is deaminated by MAO forming an aldehyde intermediate 4-hydroxyphenylacetaldehyde (4-HPAL), which can be either oxidized by ALDH generating 4-hydroxyphenylacetic acid (4-HPAA) or reduced by ALR generating Tyr [126], and Tyr can reversely return to 4-HPAL by enzyme ADH (Scheme 4).

In a similar way to that which occurs in dopamine metabolism, ethanol increases the formation of Tyr from tyramine by subsequent reduction of the previous aldehyde 4-HPAL with ALR to the Tyr and decreases its oxidation to the corresponding acid 4-HPAA. An increase in NADH/NAD+ ratio during ethanol metabolism and a competitive inhibition of ALDH by acetaldehyde have been proposed as possible mechanisms for the observed shift in tyramine oxidative metabolism [126,129,172,173]. It is important to mention that despite the lower concentration of Tyr and its precursors in virgin OO compared to HTyr, it was shown that the absorbed Tyr could be interconverted into HTyr in liver microsomes by the activity of CYP2A6 and CYP2D6, so Tyr could be a precursor of HTyr [109,174]. Neither tyrosine nor tyramine was found to generate HTyr [173].

The most abundant metabolites of Tyr were from phase-II metabolism: 4′-*O*-glucuronide and 4′-*O*-sulphate (Scheme 4). Tyr, lacking an *ortho*-diphenolic structure, could not be methylated by COMT [175]. Sulphation in the liver appears to be the major metabolic pathway of Tyr, and its metabolites may exert various biological activities in tissues. Tyr 4′-O-sulphate could protect from cell death induced by oxidized cholesterol and the biological effect of parent compounds can be retained in the metabolites [171,176]. The non-digested Tyr is biotransformed into its catabolic metabolites by gut microbiota, like HTyr (Scheme 3) [13,46,144,145].

## 5. Oleuropein

Oleuropein (Ole), the OO polyphenol with a catechol functionality (1,2-dihydroxybenzene moiety), exerts potent antioxidant activity that is widely recognized and reviewed in the literature [16,177]. Many antioxidant assays performed on Ole presented strong evidence of a free radical-scavenging activity in vitro and ex vivo [178,179]. The antioxidant and anti-inflammatory effects of Ole are considered to be in the basis of its pharmacological activities such as anticancer, cardioprotective, neuroprotective, gastroprotective, hepatoprotective, antidiabetic, antiobesity, radioprotective and other activities [177].

Many of recognized health-benefiting effects of Ole can be attributed to its anti-inflammatory activity. Indeed, the inhibition of some important inflammation mediators by Ole was observed (see Figure 1). Probably the first evidence of its anti-inflammatory effect in human cells was a decrease of IL-1β concentration in human whole blood culture, upon the addition of Ole [180]. OO extracts and Ole aglycone separately prevented stimulation of metalloproteinase 9 (MMP-9) expression and secretion in human THP-1 (monocyte-like) cells treated with TNF-α due to inhibition of NF-κB signaling [181]. An in vivo study on an experimentally induced acute colitis in mice showed decreased production of IL-1β and IL-6, NO and TNF-α, and expression of iNOS, COX-2, and MMP-9 after oral administration of Ole, principally through reduction of NF-κB activation [182]. However, in the case of chronic colitis in mice, the anti-inflammatory molecular mechanism of Ole was associated with the suppression of p38 MAPK phosphorylation which may be mediated by up-regulation of annexin A1, a protein able to block the release of inflammatory mediators [183]. The anti-inflammatory activity of Ole was demonstrated in colonic biopsies from ulcerative colitis patients and the results showed significantly lower expression of COX-2 and IL-17 in samples treated with Ole [184].

Domitrović et al. [185] demonstrated for the first time the hepatoprotective activity of Ole due to attenuation of hepatic oxidative/nitrosative stress and suppression of inflammation in CCl_4_-injured liver in vivo. The important role in prevention of acute liver damage by Ole was attributed to induction of HO-1 at least partially by up-regulation of Nrf2 transcription factor, a key regulator of cellular redox status. Recently, the protective effect of Ole against H_2_O_2_-induced apoptosis was confirmed in human liver cells where the significant increase in expression of SOD1, catalase, and glutathione peroxidase 1 was observed [186]. Ole showed renoprotective effect by attenuating cisplatin-induced acute renal injury *in vivo*, which was mediated through the inhibition of ERK signaling [187]. Also, Ole reduced apoptosis through modulating Bcl-2/Bax ratio and MAPK pathways in human embryonic kidney cells with H_2_O_2_-induced oxidative damage [188]. Investigation of anti-arthritic effects of Ole on synovial fibroblasts showed that Ole exerts anti-inflammatory and antioxidant effects via down-regulation of MAPK and NF-κB signaling pathways and induction of Nrf2-linked HO-1, controlling the production of many inflammatory mediators [189]. Oral supplementation of 10–20 mg/kg Ole reduced the airway influx of eosinophils and lymphocytes as well as IL-4 secretion in lung promoted by ovalbumin inhalation or cigarette smoke in pulmonary inflammation mice model [190].

### 5.1. Antiatherogenic and Cardioprotective Effect

Antiatherogenic effect of OOP components is well documented and confirmed by many studies [191]. Among all of them, Ole is considered to have the greatest potency as an anti-atherosclerotic agent [178]. Ole and HTyr were shown to inhibit the endothelial activation and monocytoid cell adhesion within the concentration range expected after nutritional intake from MD [192,193]. Those effects are due to their prominent antioxidant and anti-inflammatory activity, and represent possible molecular mechanisms by which Ole and other natural polyphenols may prevent early atherogenesis. The acute intervention trial on healthy volunteers who consumed olive leaf extract (51 mg Ole; 10 mg HTyr) on a single occasion demonstrated positive modulation of vascular function and IL-8 production in humans [194]. Placebo controlled randomized trial has shown that supplementation with olive leaf polyphenols (51 mg Ole and 9.7 mg HTyr daily) for 2 weeks led to increased fasting interlukin IL-6 in middle-aged over-weight men, but had no effects on IL-8, TNF-α, ultra-sensitive-CRP (us-CRP) and on carotid artery intima-media thickness [195]. A randomized, controlled trial on pre-hypertensive male who consumed a phenolic-rich olive leaf extract for 6 weeks (136 mg Ole; 6 mg HTyr) showed significantly lower BP, plasma TC, LDL cholesterol and TG relative to the control [196]. Another trial on subjects with stage-1 hypertension showed that olive leaf extract at the dosage regimen of 500 mg twice daily effectively lowered systolic and diastolic BP, with effect comparable to that of Captopril given at its effective dose (12.5–25 mg twice daily) [197]. Platelet aggregation is shown to be one of the underlying mechanisms in coronary artery disease pathogenesis. Olive phenol extracts, and especially Ole aglycone inhibited cAMP-phosphodiesterase, one of the target enzymes included in platelet aggregation [198].

Cardioprotective effect of Ole was investigated in many in vivo models. Ole reduced the release of oxidized glutathione, a sensitive marker of oxidative stress, in post-ischemic oxidative burst [199]. Rabbits treated with Ole for 3 or 6 weeks had a reduced myocardial injury after the onset of ischemia, lower cholesterol and TG levels, reduced protein carbonyl content and enhanced SOD activity [200]. Recent study suggested protective effect of Ole on myocardial ischemia/reperfusion in neonatal rat cardiomyocytes due to inhibition of apoptosis [201]. Also, Ole inhibited myocardial infarction size and decreased creatinine kinase-MB and LDH serum levels in a myocardial ischemia/reperfusion rat model [202]. Furthermore, it was shown that Ole exerts cardioprotection in cholesterol-fed animals through activation of intracellular signaling, reduces the circulating cholesterol and LDL levels and increases myocardial ATP content [203]. Ole prevented the development of myocarditis in rat model by inhibiting the cardiac MAPK and NF-κB mediated inflammatory responses [204].

### 5.2. Anticancer Effects

The anticancer effects of Ole are associated with its ability to modulate gene expression and activity of a variety of different signaling proteins that play a role in proliferation and apoptosis of cancer cells [11,43,205]. The anti-breast cancer properties of Ole have been repeatedly confirmed by vast number of studies on both estrogen receptor (ER)-positive and ER-negative breast cancer cells, demonstrating the antiproliferative and proapoptotic effects of this secoiridoid compound [205,206]. The ability of Ole to induce antimetastatic effect on human breast cancer cells due to regulation of certain genes was also confirmed [207]. Furthermore, in vitro studies sugest the significant anticancer effects of Ole on colon, liver, prostate, cervical, pancreatic, thyroid, lung, leukemia and neuroblastoma cancer cells [43,205]. In vivo study on animal model showed the inhibition of growth of MCF-7 human breast tumor xenografts and significant reduction of their invasiveness into the lung [208]. Some beneficial effects were noticed in vivo in colon, tongue, sarcoma and skin cancer animal models pointing to strong anticancer potential of Ole [205]. The recent study showed that Ole could potentiate the antitumor activity of cisplatin in human hepatocellular carcinoma (HCC) and ameliorate the side effects of cisplatin through reducing the dose required in treatment [209]. Other investigation showed protective effect of high doses of Ole (200 mg/kg) on cisplatin-induced pancreas toxicity, suggesting that Ole combined with cisplatin may improve chemotherapy efficiency in the near future [210]. Moreover, Ole demonstrated cytotoxic effect in BRAF melanoma cells and increased the cytotoxic effect of Dacarbazine and Everolimus, conventional drugs used in treatment of melanoma [211]. Overall, Ole can be used as a nutraceutical to improve the anticancer effects of current chemotherapeutics but its full therapeutic potential still remains to be determined by future animal and human studies [205].

### 5.3. Antidiabetic, Lipid-Regulating and Antiobesity Effects

Many in vivo studies on diabetic animal models already confirmed the beneficial effect of Ole or olive leaf extract rich in Ole in type 2 DM disease. Decrease in serum glucose and cholesterol levels and reduction of oxidative stress were observed in diabetic animals treated with Ole [67,212,213]. Increased glucose uptake induced by Ole, mediated through activation of AMP-activated protein kinase (AMPK) and the subsequent increase in glucose transporter (GLUT4) translocation in rat skeletal muscles was observed [214]. Furthermore, it was shown that Ole promotes glucose-stimulated insulin secretion in β-cells by stimulating the ERK/MAPK signaling pathway and inhibits the cytotoxicity induced by amylin amyloids, a hallmark feature of type 2 DM [215]. Ole was able to interfere with amylin aggregation and to prevent the cytotoxicity of amylin in pancreatic β-cells in vitro [11]. This mechanism could present a molecular basis for the beneficial effects of Ole and OOP in type 2 DM. In vivo studies suggested that olive leaf extract may attenuate insulin resistance by suppressing mRNA expression of pro-inflammatory cytokines and elevating mRNA expression of insulin receptor substrate 1 [216]. In clinical trial on patients diagnosed with type 2 DM, the treatment with olive leaf extract 500 mg daily for 14 weeks exhibited significantly lower HbA1c and fasting plasma insulin levels [217]. Also, another trial on the middle-aged overweight men showed that a 12-week supplementation with an olive leaf extract (51 mg Ole and 9.7 mg HTyr daily) was associated with an improvement in insulin sensitivity and in pancreatic β-cell responsiveness [195]. Recent trial on 20 healthy subjects revealed that Ole improves postprandial glycaemic profile via hampering NADPH oxidase 2 (Nox2)-derived oxidative stress [218].

Olive leaf extract rich in Ole, Ole aglycone and HTyr lowered serum TC, TG and LDL cholesterol levels, and increased HDL cholesterol levels, slowed down the lipid peroxidation process and enhanced antioxidant enzyme activity in Wistar rats [219]. The study on pigs treated with olive leaf supplement showed a significant decrease in plasmatic TG, lower body mass and fat storage with no significant reductions in LDL cholesterol and oxidized LDL levels [220]. In vitro studies on rat-liver cells revealed that possible lipid-lowering mechanism of olive polyphenols from OO extracts (Ole and ligstroside being most abundant) may include synergistic, rapid and direct inhibitory effect on the key regulatory enzymes involved in synthesis of fatty acid, cholesterol and TG in liver [165]. Ole exerted protective effect on progression of the nonalcoholic steatohepatitis (NASH) to fibrosis in mouse model [221]. Also, the ability of Ole to induce autophagy, a safeguard mechanism involved in intracellular protein degradation, in liver of NAFLD mice model was reported [222].

One of the major health problems nowadays is obesity, a complex disorder characterized by an increase in the number and size of adipocytes in adipose tissue, and if left untreated leads to the development of type 2 DM, cardiovascular disease and hyperlipidemia. In vitro Ole exerts anti-adipogenic effect through inhibition of both expression and activity of PPARγ, essential for the formation and function of adipocytes [223,224]. Ole was shown to be a ligand of PPARα by molecular docking simulations and the effect of Ole was associated with a significant reduction of serum TG and cholesterol levels in mice [225]. In vivo experiments suggested that Ole exerts anti-obesity effects by regulating the expression of genes involved in adipogenesis in the visceral adipose tissue of HFD-fed mice [226]. It was proposed that effect of Ole on adiposity in mice might involve a short-term mechanism, affecting the intestines and energy uptake, and a long-term homeostatic adaptation, resulting in a higher satiety [227]. Ole aglycone was shown to be an agonist of some transient receptor potential channels important for maintenance of whole body metabolism and enhanced uncoupling protein 1, a key regulator of thermogenesis in brown adipose tissue of HFD-induced obese rats [228]. Furthermore, the results of the study on Wistar rats with developed signs of metabolic syndrome suggest that an olive leaf extract containing polyphenols such as Ole and HTyr may reverse the chronic inflammation and oxidative stress and improve or normalize cardiovascular, hepatic, and metabolic signs with the exception of elevated BP [229].

### 5.4. Neuroprotective Effect

Neuroprotecive effect of Ole and other OOPs in neurodegenerative diseases was intensively investigated during the past decade and subsequently reviewed [10,11,230]. Ole was shown to have neuroprotective effects against cerebral ishemia and reperfusion injury by reducing infarct volume and neuronal apoptosis in mouse model [231]. Ole showed beneficial effects in the paraventricular nucleus of the hypothalamus in spontaneously hypertensive rats where inhibited ROS and increased the antioxidant defense system [232]. The neuroprotecive effect of Ole due to its antioxidant properties was also demonstrated in the case of cognitive dysfunction and oxidative stress induced by some anesthetic drugs in the hippocampal area of rats [233]. Also, the Ole treatment before anesthesia reduced the number of inflammatory cells and prevented degranulation in rabbits [234].

Ole aglycone hinders amyloid aggregation of Aβ(1–42) and its cytotoxicity, favoring the formation of stable harmless protofibrils, structurally different from the typical Aβ(1–42) fibrils [11]. Study on mice model of amyloid-β deposition showed the improvement of the cognitive performance in young/middle-aged mice and remarkably reduced β-amyloid levels and plaque deposits in cerebral tissue after administration of Ole aglycone. Also, brain of Ole-treated mice displayed very intense autophagic reaction, the same as in healthy wild-type mice. In vitro study confirmed the earlier hypothesis that autophagy activation by Ole proceeds via the AMPK/mTOR signaling pathway [11].

Furthermore, Ole may prevent neuronal degeneration in a cellular dopaminergic model of PD, with significant decrease in neuronal death and reduced mitochondrial production of ROS [235]. Ole aglycone exhibited anti-amyloidogenic effect in vitro by interacting with and stabilizing α-synuclein monomers, the key proteins in the pathogenesis of PD. Also, Ole aglycone reduced the cytotoxicity of α-synuclein aggregates by hindering their binding to cell membrane components and preventing the resulting oxidative damage to cells [236]. Recent study confirmed that Ole aglycone has protective effect against the protein aggregation and cytotoxicity in the case of systemic amyloidosis [237].

Ole treatment showed alleviation of neurological deficit and brain edema associated with intracerebral haemorrhage (ICH) in rat model. In addition, it preserved the blood-brain barrier structure and attenuated oxidative stress as well as ICH-induced MAPK activation in brain tissue [238]. Also, Ole improved neurological and cognitive outcomes, alleviated brain edema, increased neurotrophic factors, and reduced apoptosis of neural cells after ischemia/reperfusion injury in rat model of cerebral ischemia [239].

Ole attenuated single prolonged stress-induced cognitive impairment significantly by inhibiting the expression of pro-inflammatory mediators in the rat brain and had an anxiolytic-like effect on behavioral and biochemical symptoms in post-traumatic stress disorder rat model [240,241]. Ole was found to exhibit significant antidepressant effects in mice, probably by reducing the parameters of oxidative and nitrosative stress, but further studies are needed to investigate the precise mechanism of action [242].

### 5.5. Antimicrobial and Antiviral Effect

Ole was shown to have the high antimicrobial activity against both Gram-negative and Gram-positive bacteria, although Ole was significantly less toxic to bacterial cells than HTyr, possibly because of its glycosidic group that might render the drug unable to penetrate cell membranes or to reach the target site [83]. Ole individually exerted a strong growth inhibition effect against *Salmonella enteritidis*, but in general, better effects were reached with combination of phenols from olive leaf extract [243]. Indeed, the extracts of olive mill waste rich in phenols demonstrated broad spectrum of antibacterial activity against *Staphylococcus aureus*, *Bacillus subtilis*, *Escherichia coli* and *Pseudomonas aeruginosa* [244]. Another investigation on the activity of olive leaf extract showed specific antimicrobial activity against *Helicobacter pylori* and *Campylobacter jejuni* but did not supported the claim of a broad spectrum of antibacterial activity of this extract [245]. Ole was shown to exhibit antiviral effect on HIV-1 infection and replication by inhibiting cell-to-cell HIV-1 transmission and viral core antigen p24 production [246].

### 5.6. Osteoprotective Effect

Although the beneficial effect of olive fruit and oil consumption on bone health is well known, the underlying mechanisms are still elusive. Many cellular, animal and human studies are conducted in attempt to clarify this question, and the focus is on the activities of OOPs [247]. Ole was able to elicit protective effects on bone loss in animal model of osteopenia, probably by modulating parameters of inflammation [248]. In vitro study of the osteoblastogenesis and adipogenesis in mesenchymal stem cells (MSCs) from human bone marrow showed that Ole could prevent age-related bone loss and osteoporosis directly by enhancing the differentiation of MSCs into osteoblasts and hindering the differentiation into adipocytes and indirectly by modulating osteoclast differentiation [249]. The formation of osteoblasts in bone marrow seems to be closely associated with adipogenesis, and age-related changes in this relationship could be responsible for the progressive adiposity of bone marrow which occurs with osteoporosis. A very recent transcriptomic analysis showed that Ole could up-regulate expression of 60% of adipogenesis-repressed genes which makes Ole a potential candidate for the prevention and treatment of diseases such as obesity and osteoporosis [250]. Ole inhibited the IL-1β-induced expression of inflammatory mediators by suppressing the activation of NF-κB and MAPK signaling pathways in human OA chondrocytes indicating that Ole may serve as a potential anti-inflammatory agent in the treatment of OA [251].

### 5.7. Other Effects

The study of skin wound healing in animal model showed that Ole accelerated the reepithelization process, enhanced collagen fiber generation, and increased the blood supply to the wounded area by up-regulation of VEGF protein expression [252]. Ole was shown to inhibit the self-assembly of collagen into fibrils, presenting a new possibility in the treatment of fibrotic diseases [253]. Results from the study on MSCs, used for transplantation after ischemic vascular events, suggested that short-term priming of human MSCs with Ole before transplantation might be a promising therapeutic strategy against ischemic vascular diseases [254]. The very recent study showed that Ole can modulate angiogenesis indirectly, acting on local senescent fibroblasts by reducing NF-κB signaling and the expression of several senescence-associated secretory phenotype factors [255].

### 5.8. Metabolism

**Scheme 5:**
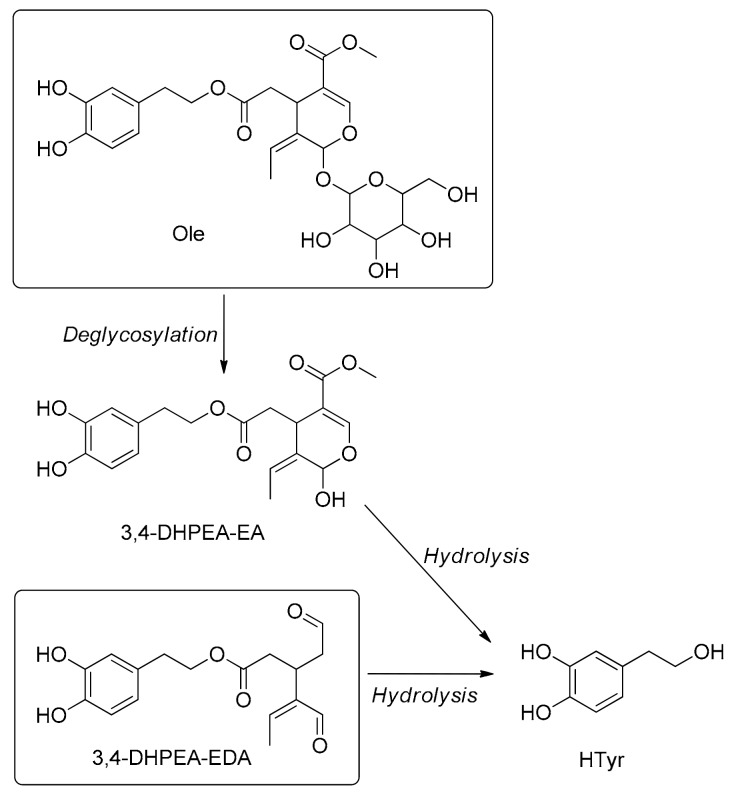
Metabolic pathways of oleuropein (Ole) and oleacein (3,4-DHPEA-EDA). 3,4-DHPEA-EA: oleuropein aglycone monoaldehyde. The catabolism of the Ole produces both PA and PP families of catabolites [46,144,145] (Scheme III).

Ole diffuses in the stomach and is stable at the gastric level during digestion (2 and 4 h after incubation in the gastric juices) [256,257]. Consequently, the bioavailability of its main metabolite HTyr is higher [44,136]. Formation of different metabolites of Ole and their distribution and concentration are strictly related to the pH of the medium and the time of permanence of the molecule in the stomach. The acid-catalyzed hydrolysis cleaves the β-glycosidic bond with the release of the aglycone moiety (3,4-DHPEA-EA) and glucose [109,258]. The Ole-aglycone formed by hydrolysis of Ole and native Ole-aglycone present in OO, together with the most abundant secoiridoid in OO oleacein, are hydrolysed into HTyr and elenolic acid and further metabolized [136] (Scheme 5). All of them are less resistant to the gastric acidic hydrolysis compared to Ole and it was shown they are more sensitive to temperature, pH and enzyme activity [136,259]. During acidic hydrolysis treatment, HTyr is released from Ole with a mean HTyr recovery of approximately 33% compared with the initial amount of Ole [151]. In the large intestine, Ole is degraded by the microflora which produces the HTyr that expresses biological activity toward large intestinal cells [43,136]. The catabolism of the Ole produces both PA and PP families of catabolites [46,144,145] (Scheme 3).

## 6. Oleocanthal

Oleocanthal (Oc), a dialdehydic derivative of decarboxymethyl elenolic acid bound to Tyr, (*p*-HPEA-EDA) was shown in vitro to inhibit both COX-1 and COX-2, inflammatory enzymes involved in the biosynthesis of inflammatory prostaglandins in a dose-dependent manner, and to be more effective than ibuprofen in inhibiting these enzymes at equimolar concentrations [260] (see Figure 1). It was also shown that Oc attenuates inflammatory mediators such as iNOS which plays a role in the pathogenesis of joint degenerative disease [261]. Oc blocks LPS-mediated inflammatory response and inhibits MMP-13 and ADAMTS-5 aggrecanase, enzyme that mediates cartilage aggrecan degradation in human primary OA chondrocytes, suggesting that Oc may be a promising agent for the treatment of inflammation in cartilage and a potential molecule to prevent disease progression [262]. The topical application of an oily fluid enriched with Oc achieves a greater reduction in post-photodynamic therapy cutaneous inflammation and a better treatment response, in comparison with the application of a conventional oily fluid [263]. Also, inhibiton of platelet aggregation by extra virgin OO was shown to be in correlation with amount of Oc [264].

Oc has been recognized as a potent pharmacological agent in the treatment of neurodegenerative disease [265,266]. Studies conducted both in vivo and in vitro have revealed the great potential of Oc in counteracting amyloid aggregation and toxicity. Protective effect of Oc against AD has been related to its ability to prevent amyloid-β (Aβ) and tau aggregation in vitro, and enhance Aβ clearance from the brain of wild-type mice in vivo. Moreover, Oc attenuated amiloid-β oligomers (Aβo)-induced inflammation, restored astrocyte neuro-supportive function by preventing Aβo-down-regulation effects on glutamine (GLT1) and glucose (GLUT1) transporters in astrocytes, and attenuated Aβo-induced synaptic protein down-regulation in neurons [267]. The neuroprotective effect of Oc against H_2_O_2_-induced oxidative stress in neuron-like SH-SY5Y cells was characterized. Oc up-regulates proteins related to the proteasome, the chaperone heat shock protein 90, the glycolytic enzyme pyruvate kinase, and the antioxidant enzyme peroxiredoxin 1 [268]. In addition to neuroprotective properties, this compound attenuates markers of inflammation implicated in AD [265,269].

Oc became a compound of interest in cancer research because of its inhibiting effect on inflammatory enzyme COX-2 that is implicated in the pathogenesis of several cancers [266]. The suppression of COX-2 and activation of AMPK in HT-29 colon cancer cells by Oc was shown [270]. Oc inhibited the proliferation, migration, and invasion of non metastatic and highly metastatic human breast and prostate cancer cell lines, mediated via inhibition of HGF-c-Met pathway, and inhibited the tumor growth in an orthotopic model of breast cancer in vivo [271,272]. c-Met inhibitory activity of Oc as well as its antimetastatic activity encouraged the development of semisynthetic bioisostere analogues possibly with even better properties [273]. Recently, a novel Oc-based c-Met inhibitor named homovanillyl sinapate was characterized [274]. Apart from c-Met inhibition, the strong antiproliferative effect of Oc against several breast cancer cell lines and downregulation of the expression of phosphorylated mTOR in metastatic breast cancer cell line was reported [275]. Oc showed a beneficial effect in suppressing growth of estrogen-dependent breast cancer cells alone and synergistically with tamoxifen, and inhibited tumor growth in animal model of estrogen-dependent breast cancer [276]. A very recent study demonstrated that Oc inhibited proliferation and migration of triple negative breast cancer cells by modulating Ca^2+^ entry through transient receptor potential channel TRPC6 while had no effect on non-tumoral breast cells [277]. In vitro antiproliferative assay on human malignant melanoma cells revealed selective activity of Oc in melanoma cells *versus* normal dermal fibroblasts in the low micromolar range of concentrations [278]. Oc could inhibit signal transducer and activator of transcription 3 (STAT3) signaling pathway, involved in apoptosis, invasion and angiogenesis of melanoma cells, and also the tumor growth and metastasis of melanoma in vivo [279]. Oc and oleacein from OO extract reduced non-melanoma skin cancer cells viability and migration, prevented colony and spheroid formation, and inhibited proliferation of atypical keratinocytes stimulated with epidermal growth factor, through the inhibition of Erk and Akt phosphorylation and the suppression of B-Raf expression [280].

In Oc treatment of human hepatocellularcarcinoma (HCC) cells the inhibition of proliferation, migration and invasion and induction of apoptosis of HCC cells were observed. In addition, suppression of tumor growth and reduction of lung metastases in an orthotopic HCC model was observed as well [281]. Oc inhibited colony formation and induced apoptosis via activation of caspases 3/7 and chromatin condensation in HCC and colorectal carcinoma cell lines. Moreover, Oc treatment induced γH2AX histone, a marker of DNA damage, increased intracellular ROS production and caused mitochondrial depolarization [282]. Oc was investigated in multiple myeloma cells with remarkable inhibition of macrophage inflammatory protein 1α, a protein crucially involved in the multiple myeloma pathogenesis [283].

Oc exerts antimicrobial activity against *Escherichia coli*, *Salmonella enterica*, *Listeria monocytogenes*, *Helicobacter pylori*, *Staphylococcus aureus* and *Enterococcus faecalis* [284].

Ligstroside and its aglycones, *p*-HPEA-EA and Oc, are subject to time-dependent hydrolysis in the acidic gastric environment in the stomach, leading to an approximately 3-fold increase in free Tyr, after only 30 min. In this case, higher amounts of Tyr may be present for absorption in the jejunum and ileum than would be expected [44]. Information on the pharmacokinetics of Oc in human is scarce – only one study has found Oc and several secoiridoid metabolites in human urine 2–6 h after the ingestion of extra virgin OO. Oc is mostly metabolized via phase I metabolism, namely hydrogenation, hydroxylation, and hydration. Some of the hydrogenated Oc metabolites are further metabolized via phase II metabolism, i.e., glucuronidation. No study was conducted to examine the bioavailability and blood–brain barrier permeability of Oc in humans [284,285]. However further studies of the metabolism in the human body are required to achieve an in-depth understanding of the metabolism and bioavailability of this compound.

## 7. Oleacein

Oleacein, dialdehydic form of decarboxymethyl elenolic acid linked to HTyr (3,4-DHPEA-EDA), has been proven to possess antioxidant and anti-inflammatory activity. Firstly, it was recognized that oleacein acts as free radical scavenger [286,287] (see Figure 1). Later it was shown that its antioxidant activity is associated with effect on the function of human neutrophils [288]. In vitro studies showed that oleacein reduces ROS production by formyl-met-leu-phenylalanine (f-MLP) and 4β-phorbol-12β-myristate-α13-acetate (PMA)-stimulated neutrophils. Stimulated neutrophils also release myeloperoxidase (MPO), which is the enzyme capable of catalyzing the formation of HOCl, a highly reactive species responsible for oxidation and chlorination reactions. Oleacein significantly reduced MPO release from neutrophils comparably with anti-inflammatory drug indomethacin. Apart from antioxidant activity, however to a lesser extent, oleacein exhibited some effects on inflammatory mediators. Oleacein enhanced the anti-inflammatory activity of human macrophages by increasing CD163 receptor expression [289]. Also it reduces the release of elastase, MMP-9 and interleukin IL-8. Moreover, it prevents the increase of CD11b/18 expression on the surface of neutrophils as well as inhibits adhesion molecules expression of VCAM-1, ICAM-1 and E-selectin, and consequently diminishes monocyte adhesion to human vascular endothelial cells [288]. It has an endothelium-dependent vasorelaxant effect mediated by an enhanced NO production, probably through a redox mechanism within endothelial cells and, at higher concentrations, an endothelium-independent vasorelaxant effect. At plasmatic concentrations, it could modulate vascular tone in vivo [290]. Oleacein possess ability to attenuate the destabilization of carotid plaque and could be potentially useful in the reduction of ischemic stroke risk. It decreases secretion of high mobility group protein-1, MMP-9, MMP-9/neutrophil gelatinase-associated lipocalin complex and tissue factor TF from the treated plaque. At the same time IL-10 and HO-1 release is increased [291]. Oleacein inhibited angiotensin converting enzyme (ACE) when tested on rabbit lungs in vitro. It is likely that oleacein inhibits ACE in an irreversible manner or acts as a tight binding inhibitor. Furthermore, it inhibits neutral endopeptidase (NEP) activity by 50%. NEP protects natriuretic peptides which are involved in the inhibition of renin-angiotensin-aldosterone system and for this reason inhibition of enzyme activity might play a role in some cardiovascular diseases [292]. It was shown that oleacein is more effective inhibitor of enzyme 5-lipoxygenase than HTyr and Oc. This might indicate the involvement of hydroxyl groups and open secoiridoid moiety of oleacein in this process. It also reduced COX-2 mRNA level in human monocytes [288].

Oleacein indicates a protective action against abdominal fat accumulation, weight gain, and liver steatosis, with improvement of insulin-dependent glucose and lipid metabolism. Protein levels of FAS, SREBP-1 and p-ERK in liver are positively modulated, indicating an improvement in liver insulin sensitivity. HFD-related hepatic insulin resistance may be partially prevented by oral administration of oleacein [293].

Oleacein exerts antimicrobial effect and the statistical correlation between oleacein concentration and Gram-positive and Gram-negative bacteria survival was found [84].

After the consumption of OO, oleacein is hydrolysed into HTyr and elenolic acid in the digestive system and further biotransformed by the enzymatic system [136]. The metabolic pathways of oleacein are shown in the Scheme 5.

## 8. Potential Clinical Applications

According to the reviewed data, HTyr and its precursors (Ole and other secoiridoid derivatives) could have the potential clinical applications in cardiovascular and neurodegenerative diseases as well as in the prevention of cancer. However, more epidemiological data are needed to support their use for this purpose [58].

The cardioprotective effects of HTyr and its precursors have been researched in numerous human intervention studies. The results of human clinical trial European Study of the Antioxidant Effects of Olive Oil and its Phenolic Compounds on lipid oxidation (EUROLIVE) [294] were one of the key reports that led the EFSA to release a claim concerning the benefits of a daily ingestion of virgin OO rich in phenolic compounds [295]. More recent clinical trials have confirmed these results, observing that plasma ox-LDL was reduced after the acute consumption of HTyr-enriched biscuits [121] or virgin OO enriched with HTyr and derivatives [32,296,297].

A large cohort of studies, such as the European Prospective Investigation into Cancer and Nutrition (EPIC) [298] and the PREvención con DIetaMEDiterránea (Prevencion con Dieta Mediterranea, PREDIMED) trials [299,300] have provided evidence that the regular consumption of OO, within the context of MD, significantly decreases the incidence of several chronic diseases such as cardiovascular, cancer, metabolic, and immune-inflammatory disorders. The results of PREDIMED trial [300] reveals a reduction of 68% in the incidence of breast cancer in women who received a diet supplemented with virgin OO. However, the observed effects in these studies cannot be attributed exclusively to HTyr and its precursors, as virgin OO contains many other phenolics and bioactive compounds [58].

The randomized clinical trial (PREDIMED) investigated HTyr or its precursors as a therapeutic tool in the prevention of neurodegenerative diseases. Results indicate that supplementation of the MedDiet with virgin OO improved the cognitive composites: frontal function (attention and executive function) and global cognition [301,302].

HTyr has been subjected to several clinical randomized trials. For example, Phase 3 of interventional study which evaluates the efficacy and tolerability of HTyr and Vitamin E in the treatment of children with biopsy-proven NASH is completed. (Trials.gov Identifier: NCT02842567) [303]. HTyr is also found in Phase 2 and 3 of trials which evaluate the effect of HTyr (25 mg orally, once daily for 1 year) on mammographic density in women at high risk of developing breast cancer (ClinicalTrials.gov Identifier: NCT02068092) [304].

Therefore, further applied research in humans is needed to verify that the discoveries generated in animal and in in vitro studies regarding the cancer prevention and neuroprotective effects of HTyr and other phenolics in OO can be translated into practical clinical recommendations [58].

## 9. Conclusions

The evidence presented in this review concerns major olive oil phenolic compounds, hydroxytyrosol, tyrosol, oleuropein, oleocanthal and oleacein, and demonstrates wide spectrum of their biological effects on physiological processes related to health and disease, as antiatherogenic, cardioprotective, anticancer, neuroprotective, antidiabetic, anti-obesity, as well as their metabolism and bioavailability. Recent research supports earlier evidence but also reveals some new aspects and therapeutic potentials. Further clinical intervention studies, as well as studies of the mechanism of their activity on molecular level, should be greatly encouraged.

## References

[1] López-Miranda J., Pérez-Jiménez F., Ros E., De Caterina R., Badimón L., Covas M.I., Escrich E., Ordovás J.M., Soriguer F., Abiá R. (2010). Olive oil and health: Summary of the II international conference on olive oil and health consensus report, Jaén and Córdoba (Spain) 2008. Nutr. Metab. Cardiovasc. Dis..

[2] Estruch R., Ros E., Salas-Salvadó J., Covas M.-I., Corella D., Arós F., Gómez-Gracia E., Ruiz-Gutiérrez V., Fiol M., Lapetra J. (2018). Primary Prevention of Cardiovascular Disease with a Mediterranean Diet Supplemented with Extra-Virgin Olive Oil or Nuts. N. Engl. J. Med..

[3] Sofi F., Macchi C., Abbate R., Gensini G.F., Casini A. (2014). Mediterranean diet and health status: An updated meta-analysis and a proposal for a literature-based adherence score. Public Health Nutr..

[4] Covas M.-I., de la Torre R., Fitó M. (2015). Virgin olive oil: A key food for cardiovascular risk protection. Br. J. Nutr..

[5] Psaltopoulou T., Kosti R.I., Haidopoulos D., Dimopoulos M., Panagiotakos D.B. (2011). Olive oil intake is inversely related to cancer prevalence: A systematic review and a meta-analysis of 13800 patients and 23340 controls in 19 observational studies. Lipids Health Dis..

[6] Buckland G., Gonzalez C.A. (2015). The role of olive oil in disease prevention: A focus on the recent epidemiological evidence from cohort studies and dietary intervention trials. Br. J. Nutr..

[7] Piroddi M., Albini A., Fabiani R., Giovannelli L., Luceri C., Natella F., Rosignoli P., Rossi T., Taticchi A., Servili M. (2017). Nutrigenomics of extra-virgin olive oil: A review. BioFactors.

[8] Menendez J.A., Joven J., Aragonès G., Barrajón-Catalán E., Beltrán-Debón R., Borrás-Linares I., Camps J., Corominas-Faja B., Cufí S., Fernández-Arroyo S. (2013). Xenohormetic and anti-aging activity of secoiridoid polyphenols present in extra virgin olive oil: A new family of gerosuppressant agents. Cell Cycle.

[9] Ghanbari R., Anwar F., Alkharfy K.M., Gilani A.-H., Saari N. (2012). Valuable Nutrients and Functional Bioactives in Different Parts of Olive (Olea europaea L.)—A Review. Int. J. Mol. Sci..

[10] Rodriguez-Morato J., Xicota L., Fito M., Farre M., Dierssen M., de la Torre R. (2015). Potential Role of Olive Oil Phenolic Compounds in the Prevention of Neurodegenerative Diseases. Molecules.

[11] Rigacci S., Stefani M. (2016). Nutraceutical Properties of Olive Oil Polyphenols. An Itinerary from Cultured Cells through Animal Models to Humans. Int. J. Mol. Sci..

[12] Parkinson L., Cicerale S. (2016). The Health Benefiting Mechanisms of Virgin Olive Oil Phenolic Compounds. Molecules.

[13] Jakobušić Brala C., Barbarić M., Karković Marković A., Uršić S., Miloš J. (2017). Biomedicinal aspects and activities of olive oil phenolic compounds. Handbook of Olive Oil. Phenolic Compounds, Production and Health Benefits.

[14] Cicerale S., Lucas L., Keast R. (2012). Antimicrobial, antioxidant and anti-inflammatory phenolic activities in extra virgin olive oil. Curr. Opin. Biotech..

[15] Fabiani R. (2016). Anti-cancer properties of olive oil secoiridoid phenols: A systematic review of in vivo studies. Food Funct..

[16] Visioli F., Poli A., Galli C. (2002). Antioxidant and other biological activities of phenols from olives and olive oil. Med. Res. Rev..

[17] Tome-Carneiro J., Crespo M.C., Iglesias-Gutierrez E., Martin R., Gil-Zamorano J., Tomas-Zapico C., Burgos-Ramos E., Correa C., Gomez-Coronado D., Lasuncion M.A. (2016). Hydroxytyrosol supplementation modulates the expression of miRNAs in rodents and in humans. J. Nutr. Biochem..

[18] Echeverria F., Ortiz M., Valenzuela R., Videla L.A. (2017). Hydroxytyrosol and Cytoprotection: A Projection for Clinical Interventions. Int. J. Mol. Sci..

[19] Robles-Almazan M., Pulido-Moran M., Moreno-Fernandez J., Ramirez-Tortosa C., Rodriguez-Garcia C., Quiles J.L., Ramirez-Tortosa MC. (2018). Hydroxytyrosol: Bioavailability, toxicity, and clinical applications. Food Res. Int..

[20] Wani T.A., Masoodi F.A., Gani A., Baba W.N., Rahmanian N., Akhter R., Wani I.A., Ahmad M. (2018). Olive oil and its principal bioactive compound: Hydroxytyrosol—A review of the recent literature. Trends Food Sci. Technol..

[21] Bayram B., Ozcelik B., Grimm S., Roeder T., Schrader C., Ernst I.M.A., Wagner A.E., Grune T., Frank J., Rimbach G. (2012). A Diet Rich in Olive Oil Phenolics Reduces Oxidative Stress in the Heart of SAMP8 Mice by Induction of Nrf2-Dependent Gene Expression. Rejuvenation Res..

[22] Scoditti E., Nestola A., Massaro M., Calabriso N., Storelli C., De Caterina R., Carluccio M.A. (2014). Hydroxytyrosol suppresses MMP-9 and COX-2 activity and expression in activated human monocytes via PKCα and PKCβ1 inhibition. Atherosclerosis.

[23] Bigagli E., Cinci L., Paccosi S., Parenti A., D’Ambrosio M., Luceri C. (2017). Nutritionally relevant concentrations of resveratrol and hydroxytyrosol mitigate oxidative burst of human granulocytes and monocytes and the production of pro-inflammatory mediators in LPS-stimulated RAW 264.7 macrophages. Int. Immunopharmacol..

[24] Perona J.S., Cabello-Moruno R., Ruiz-Gutierrez V. (2006). The role of virgin olive oil components in the modulation of endothelial function. J. Nutr. Biochem..

[25] Rietjens S.J., Bast A., Haenen G.R.M.M. (2007). New insights into controversies on the antioxidant potential of the olive oil antioxidant hydroxytyrosol. J. Agric. Food Chem..

[26] EFSA Panel on Dietetic Products, Nutrition and Allergies (NDA) (2010). Scientific Opinion on the substantiation of health claims related to vitamin E and protection of DNA, proteins and lipids from oxidative damage. EFSA J..

[27] Berrougui H., Ikhlef S., Khalil A. (2015). Extra Virgin Olive Oil Polyphenols Promote Cholesterol Efflux and Improve HDL Functionality. Evid.-Based Complement. Altern. Med..

[28] Calabriso N., Gnoni A., Stanca E., Cavallo A., Damiano F., Siculella L., Carluccio M.A. (2018). Hydroxytyrosol Ameliorates Endothelial Function under Inflammatory Conditions by Preventing Mitochondrial Dysfunction. Oxid. Med. Cell. Longev..

[29] Wu X., Li C., Mariyam Z., Jiang P., Zhou M., Zeb F., ul Haq I., Chen A., Feng Q. (2019). Acrolein-induced atherogenesis by stimulation of hepatic flavin containing monooxygenase 3 and a protection from hydroxytyrosol. J. Cell. Physiol..

[30] Fuccelli R., Fabiani R., Rosignoli P. (2018). Hydroxytyrosol Exerts Anti-Inflammatory and Anti-Oxidant Activities in a Mouse Model of Systemic Inflammation. Molecules.

[31] Colica C., Di Renzo L., Trombetta D., Smeriglio A., Bernardini S., Cioccoloni G., Costa de Miranda R., Gualtieri P., Sinibaldi Salimei P., De Lorenzo A. (2017). Antioxidant Effects of a Hydroxytyrosol-Based Pharmaceutical Formulation on Body Composition, Metabolic State, and Gene Expression: A Randomized Double-Blinded, Placebo-Controlled Crossover Trial. Oxid. Med. Cell. Longev..

[32] Crespo M.C., Tome-Carneiro J., Burgos-Ramos E., Kohen V.L., Espinosa M.I., Herranz J., Visioli F. (2015). One-week administration of hydroxytyrosol to humans does not activate Phase II enzymes. Pharmacol. Res..

[33] Catalan U., Lopez de las Hazas M.-C., Pinol C., Rubio L., Motilva M.-J., Fernandez-Castillejo S., Sola R. (2018). Hydroxytyrosol and its main plasma circulating metabolites attenuate the initial steps of atherosclerosis through inhibition of the MAPK pathway. J. Funct. Food.

[34] Gonzalez-Santiago M., Martin-Bautista E., Carrero J.J., Fonolla J., Baro L., Bartolome M.V., Gil-Loyzaga P., Lopez-Huertas E. (2006). One-month administration of hydroxytyrosol, a phenolic antioxidant present in olive oil, to hyperlipemic rabbits improves blood lipid profile, antioxidant status and reduces atherosclerosis development. Atherosclerosis.

[35] Tomé-Carneiro J., Visioli F. (2016). Polyphenol-based nutraceuticals for the prevention and treatment of cardiovascular disease: Review of human evidence. Phytomedicine.

[36] Lopez-Huertas E., Fonolla J. (2017). Hydroxytyrosol supplementation increases vitamin C levels in vivo. A human volunteer trial. Redox. Biol..

[37] Xie Y., Xu Y., Chen Z., Lu W., Li N., Wang Q., Shao L., Li Y., Yang G., Bian X. (2017). A new multifunctional hydroxytyrosol-fenofibrate with antidiabetic, antihyperlipidemic, antioxidant and antiinflammatory action. Biomed. Pharmacother..

[38] Xie Y.-D., Chen Z.-Z., Li N., Lu W.-F., Xu Y.-H., Lin Y.-Y., Shao L.-H., Wang Q.-T., Guo L.-Y., Gao Y.-Q. (2018). Hydroxytyrosol nicotinate, a new multifunctional hypolipidemic and hypoglycemic agent. Biomed. Pharmacother..

[39] Xie Y.-D., Chen Z.-Z., Shao L.-H., Wang Q.-T., Li N., Lu W.-F., Xu Y.-H., Gao Y.-Q., Guo L.-Y., Li Y.-P. (2018). A new multifunctional hydroxytyrosol-clofibrate with hypolipidemic, antioxidant, and hepatoprotective effects. Bioorg. Med. Chem. Lett..

[40] Gonzalez-Correa J.A., Navas M.D., Munoz-Marin J., Trujillo M., Fernandez-Bolanos J., Pedro de la Cruz J. (2008). Effects of hydroxytyrosol and hydroxytyrosol acetate administration to rats on platelet function compared to acetylsalicylic acid. J. Agric. Food Chem..

[41] Catalan U., Rubio L., Lopez de las Hazas M.-C., Herrero P., Nadal P., Canela N., Pedret A., Motilva M.-J., Sola R. (2016). Hydroxytyrosol and its complex forms (secoiridoids) modulate aorta and heart proteome in healthy rats: Potential cardio-protective effects. Mol. Nutr. Food Res..

[42] Fabiani R., Sepporta M.V., Rosignoli P., De Bartolomeo A., Crescimanno M., Morozzi G. (2012). Anti-proliferative and pro-apoptotic activities of hydroxytyrosol on different tumour cells: The role of extracellular production of hydrogen peroxide. Eur. J. Nutr..

[43] Imran M., Nadeem M., Gilani S.A., Khan S., Sajid M.W., Amir R.M. (2018). Antitumor Perspectives of Oleuropein and Its Metabolite Hydroxytyrosol: Recent Updates. J. Food Sci..

[44] Corona G., Tzounis X., Assunta DessÌ M., Deiana M., Debnam E.S., Visioli F., Spencer J.P.E. (2006). The fate of olive oil polyphenols in the gastrointestinal tract: Implications of gastric and colonic microflora-dependent biotransformation. Free Radic. Res..

[45] Corona G., Deiana M., Incani A., Vauzour D., Dessi M.A., Spencer J.P.E. (2009). Hydroxytyrosol inhibits the proliferation of human colon adenocarcinoma cells through inhibition of ERK1/2 and cyclin D1. Mol. Nutr. Food Res..

[46] De las Hazas M.C.L., Piñol C., Macià A., Motilva M.-J. (2017). Hydroxytyrosol and the Colonic Metabolites Derived from Virgin Olive Oil Intake Induce Cell Cycle Arrest and Apoptosis in Colon Cancer Cells. J. Agric. Food Chem..

[47] Sun L., Luo C., Liu J. (2014). Hydroxytyrosol induces apoptosis in human colon cancer cells through ROS generation. Food Funct..

[48] Terzuoli E., Giachetti A., Ziche M., Donnini S. (2016). Hydroxytyrosol, a product from olive oil, reduces colon cancer growth by enhancing epidermal growth factor receptor degradation. Mol. Nut. Food Res..

[49] Bernini R., Carastro I., Palmini G., Tanini A., Zonefrati R., Pinelli P., Brandi M.L., Romani A. (2017). Lipophilization of Hydroxytyrosol-Enriched Fractions from Olea europaea L. Byproducts and Evaluation of the in Vitro Effects on a Model of Colorectal Cancer Cells. J. Agric. Food Chem..

[50] Rosignoli P., Fuccelli R., Sepporta M.V., Fabiani R. (2016). In vitro chemo-preventive activities of hydroxytyrosol: The main phenolic compound present in extra-virgin olive oil. Food Funct..

[51] Zubair H., Bhardwaj A., Ahmad A., Srivastava S.K., Khan M.A., Patel G.K., Singh S., Singh A.P. (2017). Hydroxytyrosol Induces Apoptosis and Cell Cycle Arrest and Suppresses Multiple Oncogenic Signaling Pathways in Prostate Cancer Cells. Nutr. Cancer.

[52] Calahorra J., Martínez-Lara E., De Dios C., Siles E. (2018). Hypoxia modulates the antioxidant effect of hydroxytyrosol in MCF-7 breast cancer cells. PLoS ONE.

[53] Zhao B., Ma Y., Xu Z., Wang J., Wang F., Wang D., Pan S., Wu Y., Pan H., Xu D. (2014). Hydroxytyrosol, a natural molecule from olive oil, suppresses the growth of human hepatocellular carcinoma cells via inactivating AKT and nuclear factor-kappa B pathways. Cancer Lett..

[54] Tutino V., Caruso M.G., Messa C., Perri E., Notarnicola M. (2012). Antiproliferative, Antioxidant and Anti-inflammatory Effects of Hydroxytyrosol on Human Hepatoma HepG2 and Hep3B Cell Lines. Anticancer Res..

[55] Li S., Han Z., Ma Y., Song R., Pei T., Zheng T., Wang J., Xu D., Fang X., Jiang H. (2014). Hydroxytyrosol inhibits cholangiocarcinoma tumor growth: An in vivo and in vitro study. Oncol. Rep..

[56] Goldsmith C., Bond D., Jankowski H., Weidenhofer J., Stathopoulos C., Roach P., Scarlett C. (2018). The Olive Biophenols Oleuropein and Hydroxytyrosol Selectively Reduce Proliferation, Influence the Cell Cycle, and Induce Apoptosis in Pancreatic Cancer Cells. Int. J. Mol. Sci..

[57] Toteda G., Lupinacci S., Vizza D., Bonofiglio R., Perri E., Bonofiglio M., Lofaro D., La Russa A., Leone F., Gigliotti P. (2017). High doses of hydroxytyrosol induce apoptosis in papillary and follicular thyroid cancer cells. J. Endocrinol. Invest..

[58] De las Hazas M.C.L., Rubio L., Macia A., Motilva M.J. (2018). Hydroxytyrosol: Emerging Trends in Potential Therapeutic Applications. Curr. Pharml. Desig.

[59] Orsini F., Ami D., Lascialfari A., Natalello A. (2018). Inhibition of lysozyme fibrillogenesis by hydroxytyrosol and dopamine: An Atomic Force Microscopy study. Int. J. Biol. Macromol..

[60] Goldstein D.S., Jinsmaa Y., Sullivan P., Holmes C., Kopin I.J., Sharabi Y. (2016). 3,4-Dihydroxyphenylethanol (Hydroxytyrosol) Mitigates the Increase in Spontaneous Oxidation of Dopamine During Monoamine Oxidase Inhibition in PC12 Cells. Neurochem. Res..

[61] Hornedo-Ortega R., Cerezo A.B., Troncoso A.M., Garcia-Parrilla M.C. (2018). Protective effects of hydroxytyrosol against alpha-synuclein toxicity on PC12 cells and fibril formation. Food Chem. Toxicol..

[62] Funakohi-Tago M., Sakata T., Fujiwara S., Sakakura A., Sugai T., Tago K., Tamura H. (2017). Hydroxytyrosol butyrate inhibits 6-OHDA-induced apoptosis through activation of the Nrf2/HO-1 axis in SH-SY5Y cells. Eur. J. Pharmacol..

[63] Zheng A., Li H., Cao K., Xu J., Zou X., Li Y., Chen C., Liu J., Feng Z. (2015). Maternal hydroxytyrosol administration improves neurogenesis and cognitive function in prenatally stressed offspring. J. Nutr. Biochem..

[64] Davinelli S., Maes M., Corbi G., Zarrelli A., Willcox D.C., Scapagnini G. (2016). Dietary phytochemicals and neuroinflammaging: From mechanistic insights to translational challenges. Immun. Ageing.

[65] Priore P., Gnoni A., Natali F., Testini M., Gnoni G.V., Siculella L., Damiano F. (2017). Oleic Acid and Hydroxytyrosol Inhibit Cholesterol and Fatty Acid Synthesis in C6 Glioma Cells. Oxid. Med. Cell Longev..

[66] Carito V., Ceccanti M., Cestari V., Natella F., Bello C., Coccurello R., Mancinelli R., Fiore M. (2017). Olive polyphenol effects in a mouse model of chronic ethanol addiction. Nutrition.

[67] Jemai H., El Feki A., Sayadi S. (2009). Antidiabetic and Antioxidant Effects of Hydroxytyrosol and Oleuropein from Olive Leaves in Alloxan-Diabetic Rats. J. Agric. Food Chem..

[68] Antonio Lopez-Villodres J., Abdel-Karim M., Pedro De La Cruz J., Dolores Rodriguez-Perez M., Julio Reyes J., Guzman-Moscoso R., Rodriguez-Gutierrez G., Fernandez-Bolanos J., Antonio Gonzalez-Correa J. (2016). Effects of hydroxytyrosol on cardiovascular biomarkers in experimental diabetes mellitus. J. Nutr. Biochem..

[69] Reyes J.J., Villanueva B., Lopez-Villodres J.A., De La Cruz J.P., Romero L., Rodriguez-Perez M.D., Rodriguez-Gutierrez G., Fernandez-Bolanos J., Gonzalez-Correa J.A. (2017). Neuroprotective Effect of Hydroxytyrosol in Experimental Diabetes Mellitus. J. Agric. Food Chem..

[70] Suribabu R., Pindiprolu S.S.S., Talluri S.V., Chintamaneni P., Samidala N. (2017). Protective Effects of Hydroxytyrosol from Diabetic Peripheral Neuropathy in Rodents: Implications of Antioxidant and Anti-Inflammatory Effects. Lat. Amer. J. Pharm..

[71] Carmen Crespo M., Tome-Carneiro J., Pintado C., Davalos A., Visioli F., Burgos-Ramos E. (2017). Hydroxytyrosol restores proper insulin signaling in an astrocytic model of Alzheimer’s disease. Biofactors.

[72] Soto-Alarcon S.A., Valenzuela R., Valenzuela A., Videla L.A. (2017). Liver Protective Effects of Extra Virgin Olive Oil: Interaction between Its Chemical Composition and the Cell-signaling Pathways Involved in Protection. Endocr. Metab. Immune Disord. Drug Targets.

[73] Pirozzi C., Lama A., Simeoli R., Paciello O., Pagano T.B., Mollica M.P., Di Guida F., Russo R., Magliocca S., Canani R.B. (2016). Hydroxytyrosol prevents metabolic impairment reducing hepatic inflammation and restoring duodenal integrity in a rat model of NAFLD. J. Nutr. Biochem..

[74] Valenzuela R., Echeverria F., Ortiz M., Rincón-Cervera M.Á., Espinosa A., Hernandez-Rodas M.C., Illesca P., Valenzuela A., Videla L.A. (2017). Hydroxytyrosol prevents reduction in liver activity of Δ-5 and Δ-6 desaturases, oxidative stress, and depletion in long chain polyunsaturated fatty acid content in different tissues of high-fat diet fed mice. Lipids Health Dis..

[75] Echeverría F., Valenzuela R., Bustamante A., Álvarez D., Ortiz M., Soto-Alarcon S.A., Muñoz P., Corbari A., Videla L.A. (2018). Attenuation of High-Fat Diet-Induced Rat Liver Oxidative Stress and Steatosis by Combined Hydroxytyrosol- (HT-) Eicosapentaenoic Acid Supplementation Mainly Relies on HT. Oxid. Med. Cell Longev..

[76] Lemonakis N., Poudyal H., Halabalaki M., Brown L., Tsarbopoulos A., Skaltsounis A.-L., Gikas E. (2017). The LC-MS-based metabolomics of hydroxytyrosol administration in rats reveals amelioration of the metabolic syndrome. J. Chromatogr. B Analyt. Technol. Biomed. Life Sci..

[77] Warnke I., Goralczyk R., Fuhrer E., Schwager J. (2011). Dietary constituents reduce lipid accumulation in murine C3H10 T1/2 adipocytes: A novel fluorescent method to quantify fat droplets. Nutr. Metabol..

[78] Dagla I., Benaki D., Baira E., Lemonakis N., Poudyal H., Brown L., Tsarbopoulos A., Skaltsounis A.-L., Mikros E., Gikas E. (2018). Alteration in the liver metabolome of rats with metabolic syndrome after treatment with Hydroxytyrosol. A Mass Spectrometry And Nuclear Magnetic Resonance - based metabolomics study. Talanta.

[79] Stefanon B., Colitti M. (2016). Original Research: Hydroxytyrosol, an ingredient of olive oil, reduces triglyceride accumulation and promotes lipolysis in human primary visceral adipocytes during differentiation. Exp. Biol. Med..

[80] Wang N., Liu Y., Ma Y., Wen D. (2018). Hydroxytyrosol ameliorates insulin resistance by modulating endoplasmic reticulum stress and prevents hepatic steatosis in diet-induced obesity mice. J. Nutr. Biochem..

[81] Illesca P., Valenzuela R., Espinosa A., Echeverría F., Soto-Alarcon S., Ortiz M., Videla L.A. (2019). Hydroxytyrosol supplementation ameliorates the metabolic disturbances in white adipose tissue from mice fed a high-fat diet through recovery of transcription factors Nrf2, SREBP-1c, PPAR-γ and NF-κB. Biomed. Pharmacother..

[82] Valenzuela R., Illesca P., Echeverria F., Espinosa A., Rincon-Cervera M.A., Ortiz M., Hernandez-Rodas M.C., Valenzuela A., Videla L.A. (2017). Molecular adaptations underlying the beneficial effects of hydroxytyrosol in the pathogenic alterations induced by a high-fat diet in mouse liver: PPAR-alpha and Nrf2 activation, and NF-kappa B down-regulation. Food Funct..

[83] Bisignano G., Tomaino A., Lo Cascio R., Crisafi G., Uccella N., Saija A. (1999). On the in-vitro antimicrobial activity of oleuropein and hydroxytyrosol. J. Pharm. Pharmacol..

[84] Medina E., De Castro A., Romero C., Brenes M. (2006). Comparison of the concentrations of phenolic compounds in olive oils and other plant oils: Correlation with antimicrobial activity. J. Agric. Food Chem..

[85] Wu H., Jiang K., Zhang T., Zhao G., Deng G. (2017). Hydroxytyrosol exerts an anti-inflammatory effect by suppressing Toll-like receptor 2 and TLR 2 downstream pathways in Staphylococcus aureus-induced mastitis in mice. J. Funct. Food.

[86] Ghalandari M., Naghmachi M., Oliverio M., Nardi M., Shirazi H.R.G., Eilami O. (2018). Antimicrobial effect of Hydroxytyrosol, Hydroxytyrosol Acetate and Hydroxytyrosol Oleate on Staphylococcus Aureus and Staphylococcus Epidermidis. Elect. J. Gen. Med..

[87] Sogawa K., Kobayashi M., Suzuki J., Sanda A., Kodera Y., Fukuyama M. (2018). Inhibitory Activity of Hydroxytyrosol against Streptolysin O-Induced Hemolysis. Biocontrol. Sci..

[88] Eilami O., Oliverio M., Hosseinian S., Motlagh A.H., Naghmachi M. (2017). Antimicrobial Effects of Hydroxytyrosol Extracted From Olive Leaves, on Propionibacterium Acnes. World Family Med..

[89] Furneri P.M., Piperno A., Sajia A., Bisignano G. (2004). Antimycoplasmal activity of hydroxytyrosol. Antimicrob. Agents Chemother..

[90] Medina-Martinez M.S., Truchado P., Castro-Ibanez I., Allende A. (2016). Antimicrobial activity of hydroxytyrosol: A current controversy. Biosci. Biotechnol. Biochem..

[91] Bedoya L.M., Beltran M., Obregon-Calderon P., Garcia-Perez J., de la Torre H.E., Gonzalez N., Perez-Olmeda M., Aunon D., Capa L., Gomez-Acebo E. (2016). Hydroxytyrosol: A new class of microbicide displaying broad anti-HIV-1 activity. Aids.

[92] Yamada K., Ogawa H., Hara A., Yoshida Y., Yonezawa Y., Karibe K., Nghia V.B., Yoshimura H., Yamamoto Y., Yamada M. (2009). Mechanism of the antiviral effect of hydroxytyrosol on influenza virus appears to involve morphological change of the virus. Antiviral Resear..

[93] Diallinas G., Rafailidou N., Kalpaktsi I., Komianou A.C., Tsouvali V., Zantza I., Mikros E., Skaltsounis A.L., Kostakis I.K. (2018). Hydroxytyrosol (HT) Analogs Act as Potent Antifungals by Direct Disruption of the Fungal Cell Membrane. Front. Microbiol..

[94] Belmonte-Reche E., Martinez-Garcia M., Penalver P., Gomez-Perez V., Lucas R., Gamarro F., Maria Perez-Victoria J., Carlos Morales J. (2016). Tyrosol and hydroxytyrosol derivatives as antitrypanosomal and antileishmanial agents. Eur. J. Med. Chem..

[95] Fernandez-Pastor I., Martinez-Garcia M., Medina-O’Donnell M., Rivas F., Martinez A., Perez-Victoria J.M., Parra A. (2018). Semisynthesis of omega-Hydroxyalkylcarbonate Derivatives of Hydroxytyrosol as Antitrypanosome Agents. J. Nat. Prod..

[96] Yonezawa Y., Miyashita T., Nejishima H., Takeda Y., Imai K., Ogawa H. (2018). Anti-inflammatory effects of olive-derived hydroxytyrosol on lipopolysaccharide-induced inflammation in RAW264.7 cells. J. Vet. Med. Sci..

[97] Angeles Rosillo M., Sanchez-Hidalgo M., Castejon M.L., Montoya T., Gonzalez-Benjumea A., Fernandez-Bolanos J.G., Alarcon-de-la-Lastra C. (2017). Extra-virgin olive oil phenols hydroxytyrosol and hydroxytyrosol acetate, down-regulate the production of mediators involved in joint erosion in human synovial cells. J. Funct. Food.

[98] Cetrullo S., D’Adamo S., Guidotti S., Borzi R.M., Flamigni F. (2016). Hydroxytyrosol prevents chondrocyte death under oxidative stress by inducing autophagy through sirtuin 1-dependent and -independent mechanisms. Biochim. Biophys. Acta-Gen. Subj..

[99] Aparicio-Soto M., Sanchez-Hidalgo M., Cardeno A., Gonzalez-Benjumea A., Fernandez-Bolanos J.G., Alarcon-de-la-Lastra C. (2017). Dietary hydroxytyrosol and hydroxytyrosyl acetate supplementation prevent pristane-induced systemic lupus erythematous in mice. J. Funct. Food.

[100] Sánchez-Fidalgo S., Villegas I., Aparicio-Soto M., Cárdeno A., Rosillo Ma.Á., González-Benjumea A., Marset A., López Ó., Maya I., Fernández-Bolaños J.G. (2015). Effects of dietary virgin olive oil polyphenols: Hydroxytyrosyl acetate and 3,4-dihydroxyphenylglycol on DSS-induced acute colitis in mice. J. Nutr. Biochem..

[101] Yang X., Jing T., Li Y., He Y., Zhang W., Wang B., Zhang J., Wei J., Li R. (2017). Hydroxytyrosol Attenuates LPS-Induced Acute Lung Injury in Mice by Regulating Autophagy and Sirtuin Expression. Curr. Mol. Med..

[102] Hagiwara K., Goto T., Araki M., Miyazaki H., Hagiwara H. (2011). Olive polyphenol hydroxytyrosol prevents bone loss. Eur. J. Pharmacol..

[103] García-Martínez O., De Luna-Bertos E., Ramos-Torrecillas J., Ruiz C., Milia E., Lorenzo M.L., Jimenez B., Sánchez-Ortiz A., Rivas A. (2016). Phenolic Compounds in Extra Virgin Olive Oil Stimulate Human Osteoblastic Cell Proliferation. PLoS ONE.

[104] Bonura A., Vlah S., Longo A., Bulati M., Melis M.R., Cibella F., Colombo P. (2016). Hydroxytyrosol modulates Par j 1-induced IL-10 production by PBMCs in healthy subjects. Immunobiology.

[105] Salucci S., Burattini S., Battistelli M., Buontempo F., Canonico B., Martelli A.M., Papa S., Falcieri E. (2015). Tyrosol prevents apoptosis in irradiated keratinocytes. J. Dermatol. Sci..

[106] Avola R., Graziano A.C.E., Pannuzzo G., Bonina F., Cardile V. (2019). Hydroxytyrosol from olive fruits prevents blue-light-induced damage in human keratinocytes and fibroblasts. J. Cell Physiol..

[107] Martinez-Lara E., Pena A., Calahorra J., Canuelo A., Siles E. (2016). Hydroxytyrosol decreases the oxidative and nitrosative stress levels and promotes angiogenesis through HIF-1 independent mechanisms in renal hypoxic cells. Food Funct..

[108] Visioli F., Galli C., Bornet F., Mattei A., Patelli R., Galli G., Caruso D. (2000). Olive oil phenolics are dose-dependently absorbed in humans. FEBS Lett..

[109] De las Hazas M.C.L., Godinho-Pereira J., Macia A., Almeida A.F., Ventura M.R., Motilva M.-J., Santos C.J. (2018). Brain uptake of hydroxytyrosol and its main circulating metabolites: Protective potential in neuronal cells. J. Funct. Food.

[110] Domínguez-Perles R., Auñón D., Ferreres F., Gil-Izquierdo A. (2017). Gender differences in plasma and urine metabolites from Sprague–Dawley rats after oral administration of normal and high doses of hydroxytyrosol, hydroxytyrosol acetate, and DOPAC. Eur. J. Nutr..

[111] Bernini R., Montani M.S.G., Merendino N., Romani A., Velotti F. (2015). Hydroxytyrosol-Derived Compounds: A Basis for the Creation of New Pharmacological Agents for Cancer Prevention and Therapy. J. Med. Chem..

[112] Mateos R., Pereira-Caro G., Saha S., Cert R., Redondo-Horcajo M., Bravo L., Kroon P.A. (2011). Acetylation of hydroxytyrosol enhances its transport across differentiated Caco-2 cell monolayers. Food Chem..

[113] Pereira-Caro G., Mateos R., Shikha S., Andres M., José Luis E., Laura B., Paul A.K. (2010). Transepithelial transport and metabolism of new lipophilic ether derivatives of hydroxytyrosol by enterocyte-like Caco-2/TC7 cells. J. Agric. Food Chem..

[114] Pereira-Caro G., Madrona A., Bravo L., Espartero J.L., Alcudia F., Cert A., Mateos R. (2009). Antioxidant activity evaluation of alkyl hydroxytyrosyl ethers, a new class of hydroxytyrosol derivatives. Food Chem..

[115] Grasso S., Siracusa L., Spatafora C., Renis M., Tringali C. (2007). Hydroxytyrosol lipophilic analogues: Enzymatic synthesis, radical scavenging activity and DNA oxidative damage protection. Bioorg. Chem..

[116] Manna C., Galletti P., Maisto G., Cucciolla V., D’Angelo S., Zappia V. (2000). Transport mechanism and metabolism of olive oil hydroxytyrosol in Caco-2 cells. FEBS Lett..

[117] Pastor A., Rodríguez-Morató J., Olesti E., Pujadas M., Pérez-Mañá C., Khymenets O., Fitó M., Covas M.-I., Solá R., Motilva M.-J. (2016). Analysis of free hydroxytyrosol in human plasma following the administration of olive oil. J. Chromatogr. A.

[118] Tuck K.L., Hayball P.J., Stupans I. (2002). Structural Characterization of the Metabolites of Hydroxytyrosol, the Principal Phenolic Component in Olive Oil, in Rats. J. Agric. Food Chem..

[119] Visioli F., Galli C., Grande S., Colonnelli K., Patelli C., Galli G., Caruso D. (2003). Hydroxytyrosol Excretion Differs between Rats and Humans and Depends on the Vehicle of Administration. J. Nutr..

[120] González-Santiago M., Fonollá J., Lopez-Huertas E. (2010). Human absorption of a supplement containing purified hydroxytyrosol, a natural antioxidant from olive oil, and evidence for its transient association with low-density lipoproteins. Pharmacoll. Res..

[121] D’Angelo S., Manna C., Migliardi V., Mazzoni O., Morrica P., Capasso G., Pontoni G., Galletti P., Zappia V. (2001). Pharmacokinetics and metabolism of hydroxytyrosol, a natural antioxidant from olive oil. Drug Metab. Dispos..

[122] Fernandez-Avila C., Montes R., Castellote A.I., Chisaguano A.M., Fito M., Covas M.I., Munoz-Aguallo D., Nyyssonen K., Zunft H.J., Lopez-Sabater M.C. (2015). Fast determination of virgin olive oil phenolic metabolites in human high-density lipoproteins. Biomed. Chromatogr..

[123] Serra A., Rubió L., Borràs X., Macià A., Romero M.-P., Motilva M.-J. (2012). Distribution of olive oil phenolic compounds in rat tissues after administration of a phenolic extract from olive cake. Mol. Nutr. Food Res..

[124] Schaffer S., Asseburg H., Kuntz S., Muller W.E., Eckert G.P. (2012). Effects of Polyphenols on Brain Ageing and Alzheimer’s Disease: Focus on Mitochondria. Mol. Neurobiol..

[125] Schaffer S., Müller W.E., Eckert G.P. (2010). Cytoprotective effects of olive mill wastewater extract and its main constituent hydroxytyrosol in PC12 cells. Pharmacol. Res..

[126] Rodríguez-Morató J., Boronat A., Kotronoulas A., Pujadas M., Pastor A., Olesti E., Pérez-Mañá C., Khymenets O., Fitó M., Farré M. (2016). Metabolic disposition and biological significance of simple phenols of dietary origin: Hydroxytyrosol and tyrosol. Drug Metabol. Rev..

[127] De la Torre R., Covas M.I., Pujadas M.A., Fitó M., Farré M. (2006). Is dopamine behind the health benefits of red wine?. Eur. J. Nutr..

[128] Xu C.L., Sim M.K. (1995). Reduction of dihydroxyphenylacetic acid by a novel enzyme in the rat brain. Biochem. Pharmacol..

[129] Perez-Mana C., Farre M., Pujadas M., Mustata C., Menoyo E., Pastor A., Langohr K., de la Torre R. (2015). Ethanol induces hydroxytyrosol formation in humans. Pharmacol. Res..

[130] Davis V.E., Walsh M.J. (1970). Alcohol, amines, and alkaloids: A possible biochemical basis for alcohol addiction. Science.

[131] Davis V.E., Walsh M.J., Yamanaka Y. (1970). Augmentation of alkaloid formation from dopamine by alcohol and acetaldehyde in vitro. J. Pharmacol. Exp. Ther..

[132] Tank A.W., Weiner H. (1979). Ethanol-induced alteration of dopamine metabolism in rat liver. Biochem. Pharmacol..

[133] Marchitti S.A., Deitrich R.A., Vasiliou V. (2007). Neurotoxicity and Metabolism of the Catecholamine-Derived 3,4-Dihydroxyphenylacetaldehyde and 3,4-Dihydroxyphenylglycolaldehyde: The Role of Aldehyde Dehydrogenase. Pharmacol. Rev..

[134] Rubió L., Macià A., Valls R.M., Pedret A., Romero M.-P., Solà R., Motilva M.-J. (2012). A new hydroxytyrosol metabolite identified in human plasma: Hydroxytyrosol acetate sulphate. Food Chem..

[135] De las Hazas M.C.L., Rubió L., Kotronoulas A., de la Torre R., Solà R., Motilva M.-J. (2015). Dose effect on the uptake and accumulation of hydroxytyrosol and its metabolites in target tissues in rats. Mol. Nutr. Food Res..

[136] De las Hazas M.C.L., Piñol C., Macià A., Romero M.-P., Pedret A., Solà R., Rubió L., Motilva M.-J. (2016). Differential absorption and metabolism of hydroxytyrosol and its precursors oleuropein and secoiridoids. J. Funct. Food.

[137] De las Hazas M.C.L., Motilva M.J., Piñol C., Macià A. (2016). Application of dried blood spot cards to determine olive oil phenols (hydroxytyrosol metabolites) in human blood. Talanta.

[138] Caruso D., Visioli F., Patelli R., Galli C., Galli G. (2001). Urinary excretion of olive oil phenols and their metabolites in humans. Metabolism.

[139] Kotronoulas A., Pizarro N., Serra A., Robledo P., Joglar J., Rubió L., Hernaéz Á., Tormos C., Motilva M.J., Fitó M. (2013). Dose-dependent metabolic disposition of hydroxytyrosol and formation of mercapturates in rats. Pharmacol. Res..

[140] Wu Y.-T., Lin L.-C., Tsai T.-H. (2009). Measurement of free hydroxytyrosol in microdialysates from blood and brain of anesthetized rats by liquid chromatography with fluorescence detection. J. Chromatogr. A.

[141] Mateos R., Martínez-López S., Baeza Arévalo G., Amigo-Benavent M., Sarriá B., Bravo-Clemente L. (2016). Hydroxytyrosol in functional hydroxytyrosol-enriched biscuits is highly bioavailable and decreases oxidised low density lipoprotein levels in humans. Food Chem..

[142] Serreli G., Deiana M. (2018). Biological Relevance of Extra Virgin Olive Oil Polyphenols Metabolites. Antioxidants.

[143] García-Villalba R., Larrosa M., Possemiers S., Tomás-Barberán F.A., Espín J.C. (2014). Bioavailability of phenolics from an oleuropein-rich olive (Olea europaea) leaf extract and its acute effect on plasma antioxidant status: Comparison between pre- and postmenopausal women. Eur. J. Nutr..

[144] Mosele J.I., Martín-Peláez S., Macià A., Farràs M., Valls R.-M., Catalán Ú., Motilva M.-J. (2014). Faecal microbial metabolism of olive oil phenolic compounds: In vitro and in vivo approaches. Mol. Nutr. Food Res..

[145] Mosele J., Macià A., Motilva M.-J. (2015). Metabolic and Microbial Modulation of the Large Intestine Ecosystem by Non-Absorbed Diet Phenolic Compounds: A Review. Molecules.

[146] Ozdal T., Sela D.A., Xiao J., Boyacioglu D., Chen F., Capanoglu E. (2016). The Reciprocal Interactions between Polyphenols and Gut Microbiota and Effects on Bioaccessibility. Nutrients.

[147] Parkar S.G., Trower T.M., Stevenson D.E. (2013). Fecal microbial metabolism of polyphenols and its effects on human gut microbiota. Anaerobe.

[148] Suárez M., Valls R.M., Romero M.-P., Macià A., Fernández S., Giralt M., Solà R., Motilva M.-J. (2011). Bioavailability of phenols from a phenol-enriched olive oil. Br. J. Nutr..

[149] Chashmi N.A., Emadi S., Khastar H. (2017). Protective effects of hydroxytyrosol on gentamicin induced nephrotoxicity in mice. Biochem. Biophys. Res. Commun..

[150] Miró-Casas E., Farré Albaladejo M., Covas M.-I., Rodriguez J.O., Menoyo Colomer E., Lamuela Raventós R.M., de la Torre R. (2001). Capillary Gas Chromatography–Mass Spectrometry Quantitative Determination of Hydroxytyrosol and Tyrosol in Human Urine after Olive Oil Intake. Anal. Biochem..

[151] Miró-Casas E., Covas M.-I., Fitó M., Farré-Albadalejo M., Marrugat J., de la Torre R. (2003). Tyrosol and hydroxytyrosol are absorbed from moderate and sustained doses of virgin olive oil in humans. Eur. J. Clin. Nutr..

[152] Khymenets O., Fito M., Tourino S., Munoz-Aguayo D., Pujadas M., Torres J.L., Joglar J., Farre M., Covas M.-I., de la Torre R. (2010). Antioxidant Activities of Hydroxytyrosol Main Metabolites Do Not Contribute to Beneficial Health Effects after Olive Oil Ingestion. Drug Metab. Dispos..

[153] Giordano E., Dangles O., Rakotomanomana N., Baracchini S., Visioli F. (2015). 3-O-Hydroxytyrosol glucuronide and 4-O-hydroxytyrosol glucuronide reduce endoplasmic reticulum stress in vitro. Food Funct..

[154] Atzeri A., Lucas R., Incani A., Peñalver P., Zafra-Gómez A., Melis M.P., Pizzala R., Morales J.C., Deiana M. (2016). Hydroxytyrosol and tyrosol sulfate metabolites protect against the oxidized cholesterol pro-oxidant effect in Caco-2 human enterocyte-like cells. Food Funct..

[155] Paiva-Martins F., Silva A., Almeida V., Carvalheira M., Serra C., Rodrígues-Borges J.E., Fernandes J., Belo L., Santos-Silva A. (2013). Protective Activity of Hydroxytyrosol Metabolites on Erythrocyte Oxidative-Induced Hemolysis. J. Agric. Food Chem..

[156] Deiana M., Incani A., Rosa A., Atzeri A., Loru D., Cabboi B., Paola Melis M., Lucas R., Morales J.C., Assunta Dessì M. (2011). Hydroxytyrosol glucuronides protect renal tubular epithelial cells against H2O2 induced oxidative damage. Chem. Biol. Interact..

[157] Di Benedetto R., Varì R., Scazzocchio B., Filesi C., Santangelo C., Giovannini C., Matarrese P., D’Archivio M., Masella R. (2007). Tyrosol, the major extra virgin olive oil compound, restored intracellular antioxidant defences in spite of its weak antioxidative effectiveness. Nutr. Metab. Cardiovasc. Dis..

[158] Lee K.M., Hur J., Lee Y., Yoon B.-R., Choi S.Y. (2018). Protective Effects of Tyrosol Against Oxidative Damage in L6 Muscle Cells. Food Sci. Technol. Res..

[159] Chang C.-Y., Huang I.-T., Shih H.-J., Chang Y.-Y., Kao M.-C., Shih P.-C., Huang C.-J. (2019). Cluster of differentiation 14 and toll-like receptor 4 are involved in the anti-inflammatory effects of tyrosol. J. Funct. Food.

[160] Samuel S.M., Thirunavukkarasu M., Penumathsa S.V., Paul D., Maulik N. (2008). Akt/FOXO3a/SIRT1-Mediated Cardioprotection by n-Tyrosol against Ischemic Stress in Rat in Vivo Model of Myocardial Infarction: Switching Gears toward Survival and Longevity. J. Agric. Food Chem..

[161] Plotnikov M.B., Aliev O., Sidekhmenova A., Shamanaev A.Y., Anishchenko A.M., Fomina T., Plotnikova T.M., Arkhipov A.M. (2018). Effect of p-tyrosol on hemorheological parameters and cerebral capillary network in young spontaneously hypertensive rats. Microvasc. Res..

[162] Osipenko A.N., Plotnikova T.M., Chernysheva G.A., Smolyakova V.I. (2017). The mechanisms of neuroprotective action of p-tyrosol after the global cerebral ischemia in rats. Byulleten Sibirskoy Meditsiny.

[163] Chandramohan R., Pari L. (2016). Anti-inflammatory effects of tyrosol in streptozotocin-induced diabetic Wistar rats. J. Funct. Food.

[164] Lee H., Im S.W., Jung C.H., Jang Y.J., Ha T.Y., Ahn J. (2016). Tyrosol, an olive oil polyphenol, inhibits ER stress-induced apoptosis in pancreatic beta-cell through JNK signaling. Biochem. Biophys. Res. Commun..

[165] Priore P., Siculella L., Gnoni G.V. (2014). Extra virgin olive oil phenols down-regulate lipid synthesis in primary-cultured rat-hepatocytes. J. Nutr. Biochem..

[166] Sarna L.K., Sid V., Wang P., Siow Y.L., House J.D., Karmin O. (2016). Tyrosol Attenuates High Fat Diet-Induced Hepatic Oxidative Stress: Potential Involvement of Cystathionine β-Synthase and Cystathionine γ-Lyase. Lipids.

[167] St-Laurent-Thibault C., Arseneault M., Longpré F., Ramassamy C. (2011). Tyrosol and hydroxytyrosol, two main components of olive oil, protect N2a cells against amyloid-β-induced toxicity. Involvement of the NF-κB signaling. Curr. Alzheimer Res..

[168] Amini A., Liu M., Ahmad Z. (2017). Understanding the link between antimicrobial properties of dietary olive phenolics and bacterial ATP synthase. Int. J. Biol. Macromol..

[169] Puel C., Mardon J., Agalias A., Davicco M.-J., Lebecque P., Mazur A., Horcajada M.-N., Skaltsounis A.-L., Coxam V. (2008). Major phenolic compounds in olive oil modulate bone loss in an ovariectomy/inflammation experimental model. J. Agric. Food Chem..

[170] Cañuelo A., Gilbert-López B., Pacheco-Liñán P., Martínez-Lara E., Siles E., Miranda-Vizuete A. (2012). Tyrosol, a main phenol present in extra virgin olive oil, increases lifespan and stress resistance in Caenorhabditis elegans. Mech Ageing Dev..

[171] Lee D.-H., Kim Y.-J., Kim M., Ahn J., Ha T.-Y., Lee S., Jang Y., Jung C. (2016). Pharmacokinetics of Tyrosol Metabolites in Rats. Molecules.

[172] Tacker M., Creaven P.J., McIsaac W.M. (1970). Alteration in tyramine metabolism by ethanol. Biochem. Pharmacol..

[173] Perez-Mana C., Farré M., Rodríguez-Morató J., Papaseit E., Pujadas M., Fitó M., Robledo P., Covas M.-I., Cheynier V., Meudec E. (2015). Moderate consumption of wine, through both its phenolic compounds and alcohol content, promotes hydroxytyrosol endogenous generation in humans. A randomized controlled trial. Mol. Nutr. Food Res..

[174] Rodríguez-Morató J., Robledo P., Tanner J.-A., Boronat A., Pérez-Mañá C., Oliver Chen C.-Y., Tyndale R.F., de la Torre R. (2017). CYP2D6 and CYP2A6 biotransform dietary tyrosol into hydroxytyrosol. Food Chem..

[175] Mateos R., Goya L., Bravo L. (2005). Metabolism of the Olive Oil Phenols Hydroxytyrosol, Tyrosol, and Hydroxytyrosyl Acetate by Human Hepatoma HepG2 Cells. J. Agric. Food Chem..

[176] Muriana F.J.G., Montserrat-de la Paz S., Lucas R., Bermudez B., Jaramillo S., Morales J.C., Abia R., Lopez S. (2017). Tyrosol and its metabolites as antioxidative and anti-inflammatory molecules in human endothelial cells. Food Funct..

[177] Hassen I., Casabianca H., Hosni K. (2015). Biological activities of the natural antioxidant oleuropein: Exceeding the expectation – A mini-review. J. Funct. Food.

[178] Visioli F., Bellomo G., Galli C. (1998). Free Radical-Scavenging Properties of Olive Oil Polyphenols. Biochem. Biophys. Res. Commun..

[179] Fabiani R., Rosignoli P., De Bartolomeo A., Fuccelli R., Servili M., Montedoro G.F., Morozzi G. (2008). Oxidative DNA Damage Is Prevented by Extracts of Olive Oil, Hydroxytyrosol, and Other Olive Phenolic Compounds in Human Blood Mononuclear Cells and HL60 Cells. J. Nutr..

[180] Miles E.A., Zoubouli P., Calder P.C. (2005). Differential anti-inflammatory effects of phenolic compounds from extra virgin olive oil identified in human whole blood cultures. Nutrition.

[181] Dell’Agli M., Fagnani R., Galli G.V., Maschi O., Gilardi F., Bellosta S., Crestani M., Bosisio E., De Fabiani E., Caruso D. (2010). Olive Oil Phenols Modulate the Expression of Metalloproteinase 9 in THP-1 Cells by Acting on Nuclear Factor-κB Signaling. J. Agric. Food Chem..

[182] Giner E., Andújar I., Recio M.C., Ríos J.L., Cerdá-Nicolás J.M., Giner R.M. (2011). Oleuropein Ameliorates Acute Colitis in Mice. J. Agric. Food Chem..

[183] Giner E., Recio M.-C., Ríos J.-L., Giner R.-M. (2013). Oleuropein Protects against Dextran Sodium Sulfate-Induced Chronic Colitis in Mice. J. Nat. Prod..

[184] Larussa T., Oliverio M., Suraci E., Greco M., Placida R., Gervasi S., Marasco R., Imeneo M., Paolino D., Tucci L. (2017). Oleuropein Decreases Cyclooxygenase-2 and Interleukin-17 Expression and Attenuates Inflammatory Damage in Colonic Samples from Ulcerative Colitis Patients. Nutrients.

[185] Domitrović R., Jakovac H., Marchesi V.V., Šain I., Romić Ž., Rahelić D. (2012). Preventive and therapeutic effects of oleuropein against carbon tetrachloride-induced liver damage in mice. Pharmacol. Res..

[186] Shi C., Chen X., Liu Z., Meng R., Zhao X., Liu Z., Guo N. (2017). Oleuropein protects L-02 cells against H2O2-induced oxidative stress by increasing SOD1, GPx1 and CAT expression. Biomed. Pharmacother..

[187] Potočnjak I., Škoda M., Pernjak-Pugel E., Peršić M.P., Domitrović R. (2016). Oral administration of oleuropein attenuates cisplatin-induced acute renal injury in mice through inhibition of ERK signaling. Mol. Nutr. Food Res..

[188] Maalej A., Forte M., Bouallagui Z., Donato S., Mita L., Mita D.G., Filosa S., Crispi S., Sayadi S. (2017). Olive compounds attenuate oxidative damage induced in HEK-293 cells via MAPK signaling pathway. J. Funct. Food.

[189] Castejón M.L., Rosillo M.Á., Montoya T., González-Benjumea A., Fernández-Bolaños J.M., Alarcón-de-la-Lastra C. (2017). Oleuropein down-regulated IL-1β-induced inflammation and oxidative stress in human synovial fibroblast cell line SW982. Food Funct..

[190] Kim Y.-H., Choi Y.-J., Kang M.-K., Lee E.-J., Kim D.Y., Oh H., Kang Y.-H. (2018). Oleuropein Curtails Pulmonary Inflammation and Tissue Destruction in Models of Experimental Asthma and Emphysema. J. Agric. Food Chem..

[191] Lou-Bonafonte J.M., Arnal C., Navarro M.A., Osada J. (2012). Efficacy of bioactive compounds from extra virgin olive oil to modulate atherosclerosis development. Mol. Nutr. Food Res..

[192] Carluccio M.A., Siculella L., Ancora M.A., Massaro M., Scoditti E., Storelli C., Visioli F., Distante A., De Caterina R. (2003). Olive Oil and Red Wine Antioxidant Polyphenols Inhibit Endothelial Activation: Antiatherogenic Properties of Mediterranean Diet Phytochemicals. Arterioscler. Thromb. Vasc. Biol..

[193] Dell’Agli M., Fagnani R., Mitro N., Scurati S., Masciadri M., Mussoni L., Galli G.V., Bosisio E., Crestani M., De Fabiani E. (2006). Minor Components of Olive Oil Modulate Proatherogenic Adhesion Molecules Involved in Endothelial Activation. J. Agric. Food Chem..

[194] Lockyer S., Corona G., Yaqoob P., Spencer J.P.E., Rowland I. (2015). Secoiridoids delivered as olive leaf extract induce acute improvements in human vascular function and reduction of an inflammatory cytokine: A randomised, double-blind, placebo-controlled, cross-over trial. Br. J. Nutr..

[195] De Bock M., Derraik J.G.B., Brennan C.M., Biggs J.B., Morgan P.E., Hodgkinson S.C., Hofman P.L., Cutfield W.S. (2013). Olive (Olea europaea L.) Leaf Polyphenols Improve Insulin Sensitivity in Middle-Aged Overweight Men: A Randomized, Placebo-Controlled, Crossover Trial. PLoS ONE.

[196] Lockyer S., Rowland I., Spencer J.P.E., Yaqoob P., Stonehouse W. (2017). Impact of phenolic-rich olive leaf extract on blood pressure, plasma lipids and inflammatory markers: A randomised controlled trial. Eur. J. Nutr..

[197] Susalit E., Agus N., Effendi I., Tjandrawinata R.R., Nofiarny D., Perrinjaquet-Moccetti T., Verbruggen M. (2011). Olive (Olea europaea) leaf extract effective in patients with stage-1 hypertension: Comparison with Captopril. Phytomedicine.

[198] Dell’Agli M., Maschi O., Galli G.V., Fagnani R., Dal Cero E., Caruso D., Bosisio E. (2008). Inhibition of platelet aggregation by olive oil phenols via cAMP-phosphodiesterase. Br. J. Nutr..

[199] Manna C., Migliardi V., Golino P., Scognamiglio A., Galletti P., Chiariello M., Zappia V. (2004). Oleuropein prevents oxidative myocardial injury induced by ischemia and reperfusion. J. Nutr. Biochem..

[200] Andreadou I., Iliodromitis E.K., Mikros E., Constantinou M., Agalias A., Magiatis P., Skaltsounis A.L., Kamber E., Tsantili-Kakoulidou A., Kremastinos D.T. (2006). The Olive Constituent Oleuropein Exhibits Anti-Ischemic, Antioxidative, and Hypolipidemic Effects in Anesthetized Rabbits. J. Nutr..

[201] Zhao Q., Bai Y., Li C., Yang K., Wei W., Li Z., Pan L., Li X., Zhang X. (2017). Oleuropein Protects Cardiomyocyte against Apoptosis via Activating the Reperfusion Injury Salvage Kinase Pathway In Vitro. J. Evid.-Based Complement. Altern. Med..

[202] Jin H.-X., Zhang Y.-H., Guo R.-N., Zhao S.-N. (2018). Inhibition of MEK/ERK/STAT3 signaling in oleuropein treatment inhibits myocardial ischemia/reperfusion. Int. J. Mol. Med..

[203] Andreadou I., Benaki D., Efentakis P., Bibli S.-I., Milioni A.-I., Papachristodoulou A., Zoga A., Skaltsounis A.-L., Mikros E., Iliodromitis E. (2015). The Natural Olive Constituent Oleuropein Induces Nutritional Cardioprotection in Normal and Cholesterol-Fed Rabbits: Comparison with Preconditioning. Planta Med..

[204] Zhang J.-Y., Yang Z., Fang K., Shi Z.-L., Ren D.-H., Sun J. (2017). Oleuropein prevents the development of experimental autoimmune myocarditis in rats. Internat. Immunopharmacol..

[205] Shamshoum H., Vlavcheski F., Tsiani E. (2017). Anticancer effects of oleuropein. BioFactors.

[206] Liu L., Ahn K.S., Shanmugam M.K., Wang H., Shen H., Arfuso F., Chinnathambi A., Alharbi S.A., Chang Y., Sethi G. (2019). Oleuropein induces apoptosis via abrogating NF-κB activation cascade in estrogen receptor-negative breast cancer cells. J. Cell Biochem..

[207] Hassan Z.K., Elamin M.H., Daghestani M.H., Omer S.A., Al-Olayan E.M., Elobeid M.A., Virk P., Mohammed O.B. (2012). Oleuropein Induces Anti-metastatic Effects in Breast Cancer. Asian Pac. J. Cancer Prev..

[208] Sepporta M.V., Fuccelli R., Rosignoli P., Ricci G., Servili M., Morozzi G., Fabiani R. (2014). Oleuropein inhibits tumour growth and metastases dissemination in ovariectomised nude mice with MCF-7 human breast tumour xenografts. J. Funct. Food.

[209] Sherif I.O., Al-Gayyar M.M.H. (2018). Oleuropein potentiates anti-tumor activity of cisplatin against HepG2 through affecting proNGF/NGF balance. Life Sci..

[210] Bakir M., Geyikoglu F., Koc K., Cerig S. (2018). Therapeutic effects of oleuropein on cisplatin-induced pancreas injury in rats. J. Cancer Res. Ther..

[211] Ruzzolini J., Peppicelli S., Andreucci E., Bianchini F., Scardigli A., Romani A., la Marca G., Nediani C., Calorini L. (2018). Oleuropein, the Main Polyphenol of Olea europaea Leaf Extract, Has an Anti-Cancer Effect on Human BRAF Melanoma Cells and Potentiates the Cytotoxicity of Current Chemotherapies. Nutrients.

[212] Al-Azzawie H.F., Alhamdani M.-S.S. (2006). Hypoglycemic and antioxidant effect of oleuropein in alloxan-diabetic rabbits. Life Sci..

[213] Murotomi K., Umeno A., Yasunaga M., Shichiri M., Ishida N., Koike T., Matsuo T., Abe H., Yoshida Y., Nakajima Y. (2015). Oleuropein-Rich Diet Attenuates Hyperglycemia and Impaired Glucose Tolerance in Type 2 Diabetes Model Mouse. J. Agric. Food Chem..

[214] Alkhateeb H., Al-Duais M., Qnais E. (2018). Beneficial effects of oleuropein on glucose uptake and on parameters relevant to the normal homeostatic mechanisms of glucose regulation in rat skeletal muscle. Phytother. Res..

[215] Wu L., Velander P., Liu D., Xu B. (2017). Olive Component Oleuropein Promotes β-Cell Insulin Secretion and Protects β-Cells from Amylin Amyloid-Induced Cytotoxicity. Biochemistry.

[216] Liu Y.-N., Jung J.-H., Park H., Kim H. (2014). Olive leaf extract suppresses messenger RNA expression of proinflammatory cytokines and enhances insulin receptor substrate 1 expression in the rats with streptozotocin and high-fat diet–induced diabetes. Nutr. Res..

[217] Wainstein J., Ganz T., Boaz M., Bar Dayan Y., Dolev E., Kerem Z., Madar Z. (2012). Olive Leaf Extract as a Hypoglycemic Agent in Both Human Diabetic Subjects and in Rats. J. Med. Food.

[218] Carnevale R., Silvestri R., Loffredo L., Novo M., Cammisotto V., Castellani V., Bartimoccia S., Nocella C., Violi F. (2018). Oleuropein, a component of extra virgin olive oil, lowers postprandial glycaemia in healthy subjects. Br. J. Clin. Pharmacol..

[219] Jemai H., Bouaziz M., Fki I., El Feki A., Sayadi S. (2008). Hypolipidimic and antioxidant activities of oleuropein and its hydrolysis derivative-rich extracts from Chemlali olive leaves. Chem. Biol. Interact..

[220] Paiva-Martins F., Barbosa S., Silva M., Monteiro D., Pinheiro V., Mourão J.L., Fernandes J., Rocha S., Belo L., Santos-Silva A. (2014). The effect of olive leaf supplementation on the constituents of blood and oxidative stability of red blood cells. J. Funct. Food.

[221] Kim S.W., Hur W., Li T.Z., Lee Y.K., Choi J.E., Hong S.W., Lyoo K.-S., You C.R., Jung E.S., Jung C.K. (2014). Oleuropein prevents the progression of steatohepatitis to hepatic fibrosis induced by a high-fat diet in mice. Exp. Mol. Med..

[222] Porcu C., Sideri S., Martini M., Cocomazzi A., Galli A., Tarantino G., Balsano C. (2018). Oleuropein Induces AMPK-Dependent Autophagy in NAFLD Mice, Regardless of the Gender. Int. J. Mol. Sci..

[223] Drira R., Chen S., Sakamoto K. (2011). Oleuropein and hydroxytyrosol inhibit adipocyte differentiation in 3 T3-L1 cells. Life Sci..

[224] Svobodova M., Andreadou I., Skaltsounis A.-L., Kopecky J., Flachs P. (2014). Oleuropein as an inhibitor of peroxisome proliferator-activated receptor gamma. Genes Nutr..

[225] Malliou F., Andreadou I., Gonzalez F.J., Lazou A., Xepapadaki E., Vallianou I., Lambrinidis G., Mikros E., Marselos M., Skaltsounis A.-L. (2018). The olive constituent oleuropein, as a PPARα agonist, markedly reduces serum triglycerides. J. Nutr. Biochem..

[226] Kuem N., Song S.J., Yu R., Yun J.W., Park T. (2014). Oleuropein attenuates visceral adiposity in high-fat diet-induced obese mice through the modulation of WNT10b- and galanin-mediated signalings. Mol. Nutr. Food Res..

[227] Van der Stelt I., Hoek-van den Hil E.F., Swarts H.J.M., Vervoort J.J.M., Hoving L., Skaltsounis L., Lemonakis N., Andreadou I., van Schothorst E.M., Keijer J. (2015). Nutraceutical oleuropein supplementation prevents high fat diet-induced adiposity in mice. J. Funct. Food.

[228] Oi-Kano Y., Iwasaki Y., Nakamura T., Watanabe T., Goto T., Kawada T., Watanabe K., Iwai K. (2017). Oleuropein aglycone enhances UCP1 expression in brown adipose tissue in high-fat-diet-induced obese rats by activating β-adrenergic signaling. J. Nutr. Biochem..

[229] Poudyal H., Campbell F., Brown L. (2010). Olive Leaf Extract Attenuates Cardiac, Hepatic, and Metabolic Changes in High Carbohydrate–, High Fat–Fed Rats. J. Nutr..

[230] Casamenti F., Grossi C., Rigacci S., Pantano D., Luccarini I., Stefani M. (2015). Oleuropein Aglycone: A Possible Drug against Degenerative Conditions. In Vivo Evidence of its Effectiveness against Alzheimer’s Disease. J. Alzheimers Dis..

[231] Yu H., Liu P., Tang H., Jing J., Lv X., Chen L., Jiang L., Xu J., Li J. (2016). Oleuropein, a natural extract from plants, offers neuroprotection in focal cerebral ischemia/reperfusion injury in mice. Eur. J. Pharmacol..

[232] Sun W., Wang X., Hou C., Yang L., Li H., Guo J., Huo C., Wang M., Miao Y., Liu J. (2017). Oleuropein improves mitochondrial function to attenuate oxidative stress by activating the Nrf2 pathway in the hypothalamic paraventricular nucleus of spontaneously hypertensive rats. Neuropharmacology.

[233] Alirezaei M., Rezaei M., Hajighahramani S., Sookhtehzari A., Kiani K. (2017). Oleuropein attenuates cognitive dysfunction and oxidative stress induced by some anesthetic drugs in the hippocampal area of rats. J. Physiol. Sci..

[234] Simsek T., Erbas M., Buyuk B., Pala C., Sahin H., Altinisik B. (2018). Prevention of rocuronium induced mast cell activation with prophylactic oleuropein rich diet in anesthetized rabbits. Acta Cir. Bras..

[235] Achour I., Arel-Dubeau A.-M., Renaud J., Legrand M., Attard E., Germain M., Martinoli M.-G. (2016). Oleuropein Prevents Neuronal Death, Mitigates Mitochondrial Superoxide Production and Modulates Autophagy in a Dopaminergic Cellular Model. Int. J. Mol. Sci..

[236] Palazzi L., Bruzzone E., Bisello G., Leri M., Stefani M., Bucciantini M., Polverino de Laureto P. (2018). Oleuropein aglycone stabilizes the monomeric α-synuclein and favours the growth of non-toxic aggregates. Sci. Rep..

[237] Leri M., Oropesa-Nuñez R., Canale C., Raimondi S., Giorgetti S., Bruzzone E., Bellotti V., Stefani M., Bucciantini M. (2018). Oleuropein aglycone: A polyphenol with different targets against amyloid toxicity. Biochim. Biophys. Acta Gen. Subj..

[238] Shi J., Wu G., Zou X., Jiang K. (2017). Oleuropein protects intracerebral hemorrhage-induced disruption of blood-brain barrier through alleviation of oxidative stress. Pharmacol. Rep..

[239] Zhang W., Liu X., Li Q. (2018). Protective Effects of Oleuropein Against Cerebral Ischemia/Reperfusion by Inhibiting Neuronal Apoptosis. Med. Sci. Monit..

[240] Lee B., Shim I., Lee H., Hahm D.-H. (2018). Effect of oleuropein on cognitive deficits and changes in hippocampal brain-derived neurotrophic factor and cytokine expression in a rat model of post-traumatic stress disorder. J. Nat. Med..

[241] Lee B., Shim I., Lee H., Hahm D.-H. (2018). Oleuropein reduces anxiety-like responses by activating of serotonergic and neuropeptide Y (NPY)-ergic systems in a rat model of post-traumatic stress disorder. Anim. Cells Syst..

[242] Rabiei Z., Jahanbazi S., Alibabaei Z. (2018). Antidepressant Effects of Oleuropein in Male Mice by Forced Swim Test and Tail Suspension Test. World Fam. Med. J. Middle East. J. Fam. Med..

[243] Lee O.-H., Lee B.-Y. (2010). Antioxidant and antimicrobial activities of individual and combined phenolics in Olea europaea leaf extract. Bioresour. Bioprocess..

[244] Obied H.K., Bedgood D.R., Prenzler P.D., Robards K. (2007). Bioscreening of Australian olive mill waste extracts: Biophenol content, antioxidant, antimicrobial and molluscicidal activities. Food Chem. Toxicol..

[245] Sudjana A.N., D’Orazio C., Ryan V., Rasool N., Ng J., Islam N., Riley T.V., Hammer K.A. (2009). Antimicrobial activity of commercial Olea europaea (olive) leaf extract. J. Antimicrob. Agents.

[246] Lee-Huang S., Huang P.L., Zhang D., Lee J.W., Bao J., Sun Y., Chang Y.-T., Zhang J., Huang P.L. (2007). Discovery of small-molecule HIV-1 fusion and integrase inhibitors oleuropein and hydroxytyrosol: Part, I. Integrase inhibition. Biochem. Biophys. Res. Commun..

[247] Chin K.-Y., Ima-Nirwana S. (2016). Olives and Bone: A Green Osteoporosis Prevention Option. Int. J. Environ. Res. Public Health.

[248] Puel C., Mathey J., Agalias A., Kati-coulibaly S., Mardon J., Obled C., Davicco M.-J., Lebecque P., Horcajada M.-N., Skaltsounis A.L. (2006). Dose–response study of effect of oleuropein, an olive oil polyphenol, in an ovariectomy/inflammation experimental model of bone loss in the rat. Clin. Nutr..

[249] Santiago-Mora R., Casado-Díaz A., De Castro M.D., Quesada-Gómez J.M. (2011). Oleuropein enhances osteoblastogenesis and inhibits adipogenesis: The effect on differentiation in stem cells derived from bone marrow. Osteoporos. Int..

[250] Casado-Díaz A., Anter J., Müller S., Winter P., Quesada-Gómez J.M., Dorado G. (2017). Transcriptomic analyses of the anti-adipogenic effects of oleuropein in human mesenchymal stem cells. Food Funct..

[251] Feng Z., Li X., Lin J., Zheng W., Hu Z., Xuan J., Ni W., Pan X. (2017). Oleuropein inhibits the IL-1β-induced expression of inflammatory mediators by suppressing the activation of NF-κB and MAPKs in human osteoarthritis chondrocytes. Food Funct..

[252] Mehraein F., Sarbishegi M., Aslani A. (2014). Therapeutic Effects of Oleuropein on Wounded Skin in Young Male Balb/c Mice. Wounds.

[253] Bharathy H., Fathima N.N. (2017). Exploiting oleuropein for inhibiting collagen fibril formation. Int. J. Biol. Macromol..

[254] Ji S.T., Kim Y.-J., Jung S.Y., Kim D.Y., Kang S., Park J.H., Jang W.B., Ha J., Yun J., Kwon S.-M. (2018). Oleuropein attenuates hydrogen peroxide-induced autophagic cell death in human adipose-derived stem cells. Biochem. Biophys. Res. Commun..

[255] Margheri F., Menicacci B., Laurenzana A., Del Rosso M., Fibbi G., Cipolleschi M.G., Ruzzolini J., Nediani C., Mocali A., Giovannelli L. (2019). Oleuropein aglycone attenuates the pro-angiogenic phenotype of senescent fibroblasts: A functional study in endothelial cells. J. Funct. Food.

[256] Vissers M.N., Zock P.L., Roodenburg A.J.C., Leenen R., Katan M.B. (2002). Olive Oil Phenols Are Absorbed in Humans. J. Nutr..

[257] Gikas E., Papadopoulos N., Tsarbopoulos A. (2006). Kinetic Study of the Acidic Hydrolysis of Oleuropein, the Major Bioactive Metabolite of Olive Oil. J. Liq. Chromatogr. Relat. Technol..

[258] Carrera-González M.P., Ramírez-Expósito M.J., Mayas M.D., Martínez-Martos J.M. (2013). Protective role of oleuropein and its metabolite hydroxytyrosol on cancer. Trends Food Sci. Technol..

[259] Briante R., Patumi M., Terenziani S., Bismuto E., Febbraio F., Nucci R. (2002). Olea europaea L. Leaf Extract and Derivatives: Antioxidant Properties. J. Agric. Food Chem..

[260] Beauchamp G.K., Keast R.S.J., Morel D., Lin J., Pika J., Han Q., Lee C.-H., Smith A.B., Breslin P.A.S. (2005). Ibuprofen-like activity in extra-virgin olive oil: *Phytochemistry*. Nature.

[261] Iacono A., Gómez R., Sperry J., Conde J., Bianco G., Meli R., Gómez-Reino J.J., Smith A.B., Gualillo O. (2010). Effect of oleocanthal and its derivatives on inflammatory response induced by lipopolysaccharide in a murine chondrocyte cell line. Arthritis Rheum..

[262] Scotece M., Conde J., Abella V., Lopez V., Francisco V., Ruiz C., Campos V., Lago F., Gomez R., Pino J. (2018). Oleocanthal Inhibits Catabolic and Inflammatory Mediators in LPS-Activated Human Primary Osteoarthritis (OA) Chondrocytes Through MAPKs/NF-kappa B Pathways. Cell Phys. Biochem..

[263] Segura Palacios J.M., Blazquez Sanchez N., Rivas Ruiz F., Aguilar Bernier M., Ramirez Lopez B., Fernandez Sanchez M.E., de Troya Martin M. (2019). Topical treatment with oleocanthal extract in reducing inflammatory reactions after photodynamic therapy: A prospective quasi-experimental pilot study. Complement. Med. Res..

[264] Agrawal K., Melliou E., Li X., Pedersen T.L., Wang S.C., Magiatis P., Newman J.W., Holt R.R. (2017). Oleocanthal-rich extra virgin olive oil demonstrates acute anti-platelet effects in healthy men in a randomized trial. J. Funct. Food.

[265] Abuznait A.H., Qosa H., Busnena B.A., El Sayed K.A., Kaddoumi A. (2013). Olive-Oil-Derived Oleocanthal Enhances β-Amyloid Clearance as a Potential Neuroprotective Mechanism against Alzheimer’s Disease: In Vitro and in Vivo Studies. ACS Chem. Neurosci..

[266] Parkinson L., Keast R. (2014). Oleocanthal, a Phenolic Derived from Virgin Olive Oil: A Review of the Beneficial Effects on Inflammatory Disease. Int. J. Mol. Sci..

[267] Batarseh Y.S., Mohamed L.A., Al Rihani S.B., Mousa Y.M., Siddique A.B., El Saved K.A., Kaddoumi A. (2017). Oleocanthal ameliorates amyloid-beta oligomers’ toxicity on astrocytes and neuronal cells: In vitro studies. Neuroscience.

[268] Giusti L., Angeloni C., Barbalace M.C., Lacerenza S., Ciregia F., Ronci M., Urbani A., Manera C., Digiacomo M., Macchia M. (2018). A Proteomic Approach to Uncover Neuroprotective Mechanisms of Oleocanthal against Oxidative Stress. Int. J. Mol. Sci..

[269] Qosa H., Batarseh Y.S., Mohyeldin M.M., El Sayed K.A., Keller J.N., Kaddoumi A. (2015). Oleocanthal Enhances Amyloid-beta Clearance from the Brains of TgSwDI Mice and in Vitro across a Human Blood-Brain Barrier Model. ACS Chem. Neurosci..

[270] Khanal P., Oh W.-K., Yun H.J., Namgoong G.M., Ahn S.-G., Kwon S.-M., Choi H.-K., Choi H.S. (2011). p-HPEA-EDA, a phenolic compound of virgin olive oil, activates AMP-activated protein kinase to inhibit carcinogenesis. Carcinogenesis.

[271] Elnagar A., Sylvester P., El Sayed K. (2011). (−)-Oleocanthal as a c-Met Inhibitor for the Control of Metastatic Breast and Prostate Cancers. Planta Med..

[272] Akl M.R., Ayoub N.M., Mohyeldin M.M., Busnena B.A., Foudah A.I., Liu Y.-Y., Sayed K.A.E. (2014). Olive Phenolics as c-Met Inhibitors: (−)-Oleocanthal Attenuates Cell Proliferation, Invasiveness, and Tumor Growth in Breast Cancer Models. PLoS ONE.

[273] Busnena B.A., Foudah A.I., Melancon T., El Sayed K.A. (2013). Olive secoiridoids and semisynthetic bioisostere analogues for the control of metastatic breast cancer. Bioorg. Med. Chem..

[274] Mohyeldin M.M., Akl M.R., Ebrahim H.Y., Dragoi A.M., Dykes S., Cardelli J.A., Sayed K.A.E. (2016). The oleocanthal-based homovanillyl sinapate as a novel c-Met inhibitor. Oncotarget.

[275] Khanfar M.A., Bardaweel S.K., Akl M.R., El Sayed K.A. (2015). Olive Oil-derived Oleocanthal as Potent Inhibitor of Mammalian Target of Rapamycin: Biological Evaluation and Molecular Modeling Studies: Oleocanthal Is a Potent mTOR Inhibitor. Phytother. Res..

[276] Ayoub N.M., Siddique A.B., Ebrahim H.Y., Mohyeldin M.M., El Sayed K.A. (2017). The olive oil phenolic (-)-oleocanthal modulates estrogen receptor expression in luminal breast cancer in vitro and in vivo and synergizes with tamoxifen treatment. Eur. J. Pharmacol..

[277] Diez-Bello R., Jardin I., Lopez J.J., El Haouari M., Ortega-Vidal J., Altarejos J., Salido G.M., Salido S., Rosado J.A. (2019). (-)-Oleocanthal inhibits proliferation and migration by modulating Ca2+ entry through TRPC6 in breast cancer cells. Biochim. Biophys. Acta Mol. Cell Res..

[278] Fogli S., Arena C., Carpi S., Polini B., Bertini S., Digiacomo M., Gado F., Saba A., Saccomanni G., Breschi M.C. (2016). Cytotoxic Activity of Oleocanthal Isolated from Virgin Olive Oil on Human Melanoma Cells. Nutr. Cancer.

[279] Gu Y., Wang J., Peng L. (2017). (−)-Oleocanthal exerts anti-melanoma activities and inhibits STAT3 signaling pathway. Oncol. Rep..

[280] Polini B., Digiacomo M., Carpi S., Bertini S., Gado F., Saccomanni G., Macchia M., Nieri P., Manera C., Fogli S. (2018). Oleocanthal and oleacein contribute to the in vitro therapeutic potential of extra virgin oil-derived extracts in non-melanoma skin cancer. Oxicol. In Vitro.

[281] Pei T., Meng Q., Han J., Sun H., Li L., Song R., Sun B., Pan S., Liang D., Liu L. (2016). (-)-Oleocanthal inhibits growth and metastasis by blocking activation of STAT3 in human hepatocellular carcinoma. Oncotarget..

[282] Cusimano A., Balasus D., Azzolina A., Augello G., Emma M.R., Di Sano C., Gramignoli R., Strom S.C., McCubery J.A., Montalto G. (2017). Oleocanthal exerts antitumor effects on human liver and colon cancer cells through ROS generation. Int. J. Oncol..

[283] Scotece M., Gómez R., Conde J., Lopez V., Gómez-Reino J.J., Lago F., Smith A.B., Gualillo O. (2013). Oleocanthal Inhibits Proliferation and MIP-1 Expression in Human Multiple Myeloma Cells. Curr. Med. Chem..

[284] Pang K.-L., Chin K.-Y. (2018). The Biological Activities of Oleocanthal from a Molecular Perspective. Nutrients.

[285] García-Villalba R., Carrasco-Pancorbo A., Oliveras-Ferraros C., Vázquez-Martín A., Menéndez J.A., Segura-Carretero A., Fernández-Gutiérrez A. (2010). Characterization and quantification of phenolic compounds of extra-virgin olive oils with anticancer properties by a rapid and resolutive LC-ESI-TOF MS method. J. Pharm. Biomed. Anal..

[286] Paiva-Martins F., Santos V., Mangericão H., Gordon M.H. (2006). Effects of Copper on the Antioxidant Activity of Olive Polyphenols in Bulk Oil and Oil-in-Water Emulsions. J. Agric. Food Chem..

[287] Czerwińska M., Kiss A.K., Naruszewicz M. (2012). A comparison of antioxidant activities of oleuropein and its dialdehydic derivative from olive oil, oleacein. Food Chem..

[288] Naruszewicz M., Czerwinska M.E., Kiss A.K. (2015). Oleacein. Translation from Mediterranean Diet to Potential Antiatherosclerotic Drug. Curr. Pharm. Des..

[289] Filipek A., Czerwinska M.E., Kiss A.K., Wrzosek M., Naruszewicz M. (2015). Oleacein enhances anti-inflammatory activity of human macrophages by increasing CD163 receptor expression. Phytomedicine.

[290] Segade M., Bermejo R., Silva A., Paiva-Martins F., Gil-Longo J., Campos-Toimil M. (2016). Involvement of endothelium in the vasorelaxant effects of 3,4-DHPEA-EA and 3,4-DHPEA-EDA, two major functional bioactives in olive oil. J. Funct. Food.

[291] Filipek A., Czerwinska M.E., Kiss A.K., Polanski J.A., Naruszewicz M. (2017). Oleacein may inhibit destabilization of carotid plaques from hypertensive patients. Impact on high mobility group protein-1. Phytomedicine.

[292] Czerwińska M.E., Kiss A.K., Naruszewicz M. (2014). Inhibition of human neutrophils NEP activity, CD11b/CD18 expression and elastase release by 3,4-dihydroxyphenylethanol-elenolic acid dialdehyde, oleacein. Food Chem..

[293] Lombardo G.E., Lepore S.M., Morittu V.M., Arcidiacono B., Colica C., Procopio A., Maggisano V., Bulotta S., Costa N., Mignogna C. (2018). Effects of Oleacein on High-Fat Diet-Dependent Steatosis, Weight Gain, and Insulin Resistance in Mice. Front. Endocrinol..

[294] Cicero A.F.G., Nascetti S., López-Sabater M.C., Elosua R., Salonen J.T., Nyyssönen K., Poulsen H.E., Zunft H.J., Kiesewetter H., de la Torre K. (2008). Changes in LDL fatty acid composition as a response to olive oil treatment are inversely related to lipid oxidative damage: The EUROLIVE study. J. Am. Coll. Nutr..

[295] (2011). Scientific Opinion on the substantiation of health claims related to polyphenols in olive and protection of LDL particles from oxidative damage (ID 1333, 1638, 1639, 1696, 2865), maintenance of normal blood HDL cholesterol concentrations (ID 1639), mainte. EFSA J..

[296] Valls R.-M., Farràs M., Suárez M., Fernández-Castillejo S., Fitó M., Konstantinidou V., Fuentes F., López-Miranda J., Giralt M., Covas M. (2015). Effects of functional olive oil enriched with its own phenolic compounds on endothelial function in hypertensive patients. A randomised controlled trial. Food Chem..

[297] Hohmann C.D., Cramer H., Michalsen A., Kessler C., Steckhan N., Choi K., Dobos G. (2015). Effects of high phenolic olive oil on cardiovascular risk factors: A systematic review and meta-analysis. Phytomedicine.

[298] Buckland G., Travier N., Agudo A., Fonseca-Nunes A., Navarro C., Lagiou P., Demetriou C., Amiano P., Dorronsoro M., Chirlaque M.D. (2012). Olive oil intake and breast cancer risk in the Mediterranean countries of the European Prospective Investigation into Cancer and Nutrition study. Int. J. Cancer.

[299] Medina-Remon A., Casas R., Tressserra-Rimbau A., Ros E., Martinez-Gonzalez M.A., Fito M., Corella D., Salas-Salvadó J., Lamuela-Raventos R.M., Estruch R. (2017). Polyphenol intake from a Mediterranean diet decreases inflammatory biomarkers related to atherosclerosis: A substudy of the PREDIMED trial. Bri. J. Clin. Pharmacol..

[300] Toledo E., Salas-Salvadó J., Donat-Vargas C., Buil-Cosiales P., Estruch R., Ros E., Corella D., Fitó M., Hu F.B., Arós F. (2015). Mediterranean diet and invasive breast cancer risk among women at high cardiovascular risk in the PREDIMED trial. JAMA Intern. Med..

[301] Valls-Pedret C., Sala-Vila A., Serra-Mir M., Corella D., de la Torre R., Martínez-González M.Á., Martínez-Lapiscina E.H., Fitó M., Pérez-Heras A., Salas-Salvadó J. (2015). Mediterranean diet and age-related cognitive decline. JAMA Intern. Med..

[302] Martínez-Lapiscina E.H., Clavero P., Toledo E., San Julián B., Sanchez-Tainta A., Corella D., Lamuela-Raventós R.M., Martínez J.A., Martínez-Gonzalez M.Á. (2013). Virgin olive oil supplementation and long-term cognition: The PREDIMEDNAVARRA randomized, trial. J. Nutr. Health Aging..

[303] ClinicalTrials.gov. https://clinicaltrials.gov/ct2/show/NCT02842567.

[304] ClinicalTrials.gov. https://clinicaltrials.gov/ct2/show/NCT02068092.

